# Molecular mechanisms underlying TXNIP’s anti-tumor role in breast cancer, including interaction with a novel, pro-tumor partner: CAST

**DOI:** 10.1038/s41419-025-07566-4

**Published:** 2025-04-02

**Authors:** Jasvinder Singh, Bindeshwar Sah, Yibin Deng, Robert Clarke, Liang Liu

**Affiliations:** 1 The Hormel Institute, University of Minnesota, Austin, MN 55912 USA; 2 Department of Urology, University of Minnesota Medical School, Minneapolis, MN 55455 USA; 3 Masonic Cancer Center, University of Minnesota, Minneapolis, MN 55455 USA; 4 Department of Biochemistry, Molecular Biology and Biophysics, Minneapolis, MN 55455 USA

**Keywords:** Breast cancer, Oncogenes

## Abstract

Thioredoxin-interacting protein (TXNIP) plays a pivotal role in glucose metabolism and redox signaling. Its emerging function as a potent suppressor of cell proliferation in various cancer contexts underscores its importance in cancer development. In a previous study, we found TXNIP activation by UNC0642, an inhibitor of histone methyltransferase G9A, significantly inhibited MDA-MB-231 breast cancer cell proliferation in vitro and tumor growth in vivo. Here, we demonstrated that TXNIP knockdown increased MDA-MB-231 tumor growth and metastasis in a mouse model. Reintroducing TXNIP into TXNIP-deficient HCC-1954 breast cancer cells decreased cell proliferation and migration while boosting the generation of reactive oxygen species, alongside reductions in mitochondrial respiration, mitochondrial membrane potential, and glycolysis. To elucidate the mechanisms underlying TXNIP’s antitumor effects in breast cancer cells, we conducted co-immunoprecipitation and proteomic analyses that revealed calpastatin (CAST) as a novel TXNIP-interacting protein in MDA-MB-231 and HCC-1954 cells. Overexpression of CAST, an endogenous inhibitor of calpains, significantly increased xenograft tumor growth for both MDA-MB-231 and HCC-1954 cells, underscoring its novel role as a tumor promoter. In addition, we identified a positive correlation between the expression of TXNIP and interleukin-24 (IL-24), a molecule that induces cancer-specific apoptosis in several breast cancer cell lines. Our findings also show TXNIP’s ability to decrease activation of STAT3, a key driver of oncogenesis. Finally, cells with high levels of TXNIP expression displayed increased susceptibility to IL-24 and WP1066, a specific STAT3 inhibitor, suggesting possible predictive value for TXNIP. Collectively, these findings unveil novel TXNIP-dependent pathways that may contribute to breast cancer pathogenesis, enriching our understanding of this molecule’s intricate role in cancer and potentially paving the way for clinical translation.

## Introduction

TXNIP is a versatile protein that plays a significant role in metabolic regulation and other cellular processes including apoptosis. A potent negative regulator of glucose uptake and aerobic glycolysis [1, 2], TXNIP is widely studied in diabetes research [3]. Emerging evidence also supports a role as a tumor suppressor, facilitated by TXNIP’s ability to inhibit glucose uptake and aerobic glycolysis [4]. In addition, TXNIP regulates cellular redox homeostasis and reactive oxygen species (ROS) signaling by binding to and inhibiting thioredoxin [5]. According to The Cancer Genome Atlas, TXNIP mRNA expression is frequently downregulated in human breast cancer (BC), lung squamous cell carcinoma, colon adenocarcinoma, and many other cancers relative to the corresponding normal tissues [6]. Pronounced TXNIP downregulation is associated with diminished overall survival in patients with BC, with the strongest effects observed in individuals with HER2-positive and triple-negative BC (TNBC) subtypes [7–9]. TXNIP downregulation can result from epigenetic silencing by DNA methylation and by histone modification, and transcriptional repression mediated by several transcription factors [10–12]. Evidence from both animal models and human studies suggests an antitumor function in various cancer types. For example, TXNIP-null mice exhibit a 40% higher incidence of hepatocellular carcinoma than wild-type (WT) mice [13]. Reduced TXNIP expression in T-cell lymphoma patients is linked to disease progression [14].

In light of TXNIP’s antitumor function and frequent loss in human cancers, previous studies have explored various approaches to activating TXNIP expression in cancer cells. Vitamin D3 stimulates TXNIP expression in hepatocellular carcinoma and endometrial cancer cells, coupled with induction of apoptotic cell death and ROS production [15, 16]. Epigenetic activation of TXNIP by inhibiting histone methylation and deacetylation has shown promising results in preclinical studies [11, 17]. Epigenetic drugs, such as those using the demethylating agent 5-azacytidine, histone deacetylase (HDAC) inhibitor vorinostat, or the histone methylation inhibitor 3-deazaneplanocin A, also activate TXNIP expression in vitro [12, 18–20]. These studies underscore TXNIP’s potential as a target for cancer therapy. To enable effective targeting of TXNIP, further research is warranted to unravel the intricate network of TXNIP-dependent signaling pathways involved in cancer growth and progression.

Previously, we investigated the anticancer activity of an epigenetic compound UNC0642 and its mechanism of action in suppressing breast cancer (BC) cell growth and survival [18]. UNC0642 was developed as a selective inhibitor of G9a that is responsible for histone H3K9 methylation [21]. To identify additional UNC0642 targets, we performed RNA-seq analyses in BC cells following UNC0642 treatment. UNC0642 significantly upregulated TXNIP mRNA and protein expression [18]. We further showed that TXNIP upregulation was associated with an increase in reactive oxygen species (ROS), concurrent with loss of mitochondrial membrane potential (MMP) and activation of caspase-3-dependent apoptosis [18]. These findings prompted further investigation into TXNIP’s role in BC.

In this study, we explored the mechanism underlying TXNIP’s tumor suppression activities using two ER/PR negative BC models - MDA-MB-231 (TNBC; high TXNIP expression) and HCC-1954 (HER2-positive; low TXNIP expression) [22]. TXNIP knockdown increased MDA-MB-231 tumor growth and metastasis. Reintroducing TXNIP into TXNIP-deficient HCC-1954 cells decreased cell proliferation and migration, increased the generation of reactive oxygen species, and reduced mitochondrial respiration, mitochondrial membrane potential, and glycolysis. We also identified a new TXNIP-interacting protein, calpastatin (CAST), and showed that TXNIP upregulated IL-24 while downregulated STAT3 signaling in both MDA-MB-231 and HCC-1954 BC cells.

## Materials and methods

### Cell lines and cell culture

The human BC cell lines MDA-MB-231 and HCC-1954 were maintained in RPMI medium (Cytiva) supplemented with 10% fetal bovine serum (Gibco Laboratories) and 1x penicillin-streptomycin (Sigma-Aldrich). CRISPR-Cas9-based TXNIP-knockout (TXNIP-KO) MDA-MB-231 cells were generated by Synthego. Single-cell clones were obtained by serial dilution. All cells were grown at 37 °C within a humidified 95% air:5% CO_2_ atmosphere.

### Lentivirus-mediated TXNIP and CAST knockdown and overexpression

To achieve TXNIP and CAST knockdown (KD), MDA-MB-231 and HCC-1954 cells were transduced with lentivirus expressing either scrambled shRNA (control) or shRNAs targeting either TXNIP or CAST (Vector Builder). To achieve TXNIP and CAST overexpression (OE), MDA-MB-231 and HCC-1954 cells were transduced with lentivirus expressing either mCherry (control) or TXNIP or CAST (Vector Builder). Following lentiviral infection, cells were selected using puromycin (0.7–1 µg/mL) to generate stable cell lines for subsequent in vitro and xenograft experiments.

### IncuCyte-based cell proliferation assay

Cell proliferation was measured using the IncuCyte live cell analysis system (Sartorius). Initially, cells were seeded into 96-well plates and allowed to incubate overnight to ensure cell attachment. Subsequently, the plates were transferred to an IncuCyte ZOOM platform for analysis. IncuCyte ZOOM captures images at regular intervals to measure cell confluency over the specified incubation period and determine the rate of cell growth and proliferation.

### Wound healing

BC cells were seeded in a 96-well plate and allowed to attach. A scratch wound was created in the cell monolayer using Wound Maker 96 (Essen Biosciences) to generate a uniform scratch wound in the cell monolayer. After incubation for 30 h, during which time cells migrated and invaded the wounded area, images of the scratched area were captured and measured using the live cell image analysis software (v2020C).

### Colony formation and anchorage-independent cell growth assay

BC cells were seeded in culture plates. Upon visible colony formation, cells were fixed with 4% paraformaldehyde and stained with crystal violet. Subsequent imaging was performed using a Zeiss stereo microscope; ImageJ software was used to quantify and characterize colony features. Cells were seeded at appropriate densities in six-well plates layered with supportive agarose matrices and incubated to allow colony formation. Subsequently, colony images were acquired using a Zeiss microscope equipped with apotome technology, facilitating high-resolution visualization, and colony size was quantified using ImageJ software.

### Xenograft experiments in mice

NSG mice (NOD SCID IL2Rg^null^), obtained from The Jackson Laboratory (Stock #005557), were housed in a facility at The Hormel Institute accredited by the Association for Assessment and Accreditation of Laboratory Animal Care. All animal experiments were conducted according to protocols approved by the University of Minnesota’s Institutional Animal Care and Use Committee (Protocol Number: 2111-39576A) and fully consistent with NIH guidelines for vertebrate animal care. In brief, 1 ×10^6^ BC cells (TXNIP-KD MDA-MB-231, CAST-KD MDA-MB-231, CAST-OE MDA-MB-231, TXNIP-OE HCC-1954, CAST-OE HCC-1954, and CAST-KD_TXNIP-OE HCC-1954 cell lines) suspended in a cell culture media and matrigel mixture (1:1 ratio) were injected into the mammary fat pads of NOD SCID mice. Each injection consisted of a 100 µl cell mixture. Tumor growth was monitored weekly and recorded until tumors reached a diameter of 2 cm, at which point animals were euthanized for tumor collection and subsequent analysis.

### Western blotting and immunofluorescence staining

For western blotting, cells were lysed on ice for 30 min in RIPA buffer (Thermo Fisher Scientific) with protease inhibitors. After lysis, the supernatant was collected, and protein concentration was determined using a bicinchoninic acid (BCA) assay kit (Thermo Fisher Scientific). Protein samples were denatured at 98 °C for 5 min, separated on 4–20% SDS-PAGE at 120 V for 1.5 h, and transferred to PVDF membranes at 100 V for 1 h. Membranes were blocked with skim milk, incubated overnight at 4 °C with primary antibodies, washed with tris-buffered saline–tween (TBS-T) solution, and incubated for 1 h at room temperature with HRP-conjugated secondary antibodies. TXNIP, CAST, p-STAT3, and STAT3 antibodies were purchased from Cell Signaling Technology. IL-24 antibody was purchased from Abcam. Beta-actin (loading control) was purchased from Santa Cruz. Protein bands were visualized using a chemiluminescent super signal west dura (Thermo Fisher Scientific) and imaged with an Amersham Imager 600.

For immunofluorescence (IF) staining, tissue samples were fixed in 2% paraformaldehyde (PFA), submerged in 30% sucrose solution in PBS (Cytiva), and embedded in an optimal cutting temperature compound. Cultured cells were fixed with methanol. Frozen tissue sections were sliced to 5–7 µm on glass slides using a cryostat, washed with PBS, and blocked to prevent nonspecific binding. Both tissue samples and cultured cells were incubated overnight at 4 °C with primary antibodies, then washed and incubated for 1 h with Alexa Fluor–conjugated secondary antibodies at room temperature. Finally, samples were mounted with an antifade medium containing DAPI to stain nuclei and imaged using a confocal microscope with an Airyscan detector (Zeiss LSM900) for high-resolution visualization.

### Detection of reactive oxygen species with CellROX green dye

ROS levels were measured using CellROX (Thermo Fisher Scientific), a photostable dye that emits bright green fluorescence upon oxidation. Cells were seeded in 96-well plates and incubated with 5 µM CellROX dye at 37 °C for 30 min. After incubation, cells were washed with PBS to remove excess dye. Images were acquired using a Zeiss LSM 900 confocal microscope with appropriate fluorescence filters.

### Mitochondrial membrane potential assay

Cells were seeded in a six-well plate and allowed to adhere. After incubation, cells were treated with tetramethyl rhodamine (TMRM; Thermo Fisher Scientific), a fluorescent dye that measures mitochondrial membrane potential. Following a 30-min incubation with TMRM, cells were washed with PBS to remove excess dye. Cells were then detached using trypsin and collected for analysis. The fluorescence signal emitted by the TMRM dye in the collected cells was analyzed using a BD LSR Fortessa flow cytometer and FlowJo software.

### Seahorse cell mito stress assay

Cells were seeded in a Seahorse XF-96 cell culture microplate (Agilent) to monitor their oxygen consumption rate, a key indicator of cellular bioenergetics. The resulting data were analyzed using Wave desktop software. To ensure accurate measurement of oxygen consumption, sensor cartridges were incubated overnight at 37 °C in a CO2-free humidified environment with the provided calibrant solution. On the day of the assay, the Seahorse XF-96 cell culture microplate was washed twice and incubated with Seahorse XF-RPMI media to prepare the cells for analysis. After a 1-hour equilibration period, the sensor cartridge and cell culture microplate were loaded into the XF-96 analyzer. The bioenergetic profile of BC cells was evaluated by sequentially injecting specific compounds into the wells of the microplate: oligomycin to inhibit ATP synthase and assess ATP-linked respiration, carbonyl cyanide-p-trifluoromethoxyphenylhydrazone (FCCP) to uncouple mitochondrial respiration and measure maximal respiration, and antimycin to block mitochondrial electron transport and serve as a negative control.

### Mitochondrial morphology by confocal microscopy

Cells were seeded onto chamber slides and incubated with MitoTracker dye (Thermo Fisher Scientific) for 30 min to label mitochondria. After staining, cells were washed to remove excess dye, fixed in 2% PFA, permeabilized with Triton-X and then blocked with bovine serum albumin to prevent non-specific binding. Cells were incubated overnight at 4 °C with the primary antibody against TXNIP. After washing with PBS, cells were incubated with fluorescently labeled goat anti-rabbit secondary antibody and washed again to remove excess secondary antibody. Cells were then mounted with DAPI-containing media to stain nuclei. Finally, MitoTracker-stained mitochondria and TXNIP labeled by the antibody were imaged using the Zeiss LSM 900 confocal microscope.

### Cell cycle analysis and apoptosis detection using flow cytometry

For cell cycle analysis, cells were seeded and allowed to incubate for 24 h. Cells were then trypsinized, washed, and fixed at −20 °C for 20 min. After fixation, cells were treated with RNase and incubated at 37 °C for 30 min. Propidium iodide (PI) was added, and cells were analyzed using flow cytometry to measure cell cycle distribution. For the annexin V apoptosis assay, cells were harvested, washed with PBS, and suspended in a binding buffer containing annexin V-FITC (BD Biosciences) and PI. Stained cells were analyzed using flow cytometry with a FACS Calibur to detect apoptosis by identifying cells positive for PI and subsequently positive for annexin V.

### Immunoprecipitation and mass spectrometry

For the immunoprecipitation (IP) assay, cells were pelleted and resuspended in IP lysis buffer containing a protease inhibitor cocktail, then incubated for 30 min at 4 °C with gentle agitation every 10 min to ensure thorough mixing. The cell lysate was then centrifuged at 12,000 rpm for 15 min at 4 °C to collect soluble proteins. Protein concentration was determined using the BCA method. Approximately 500 µg proteins were separated on a 4–15% gel, transferred to a PVDF membrane, and incubated overnight at 4 °C with constant rotation with anti-TXNIP or anti-CAST antibodies (1 µg per sample), or IgG (2 µg per sample) as control for IP specificity. Following antibody incubation, 50 µl sepharose agarose beads were added to the protein-antibody complexes and the mixtures were incubated for an additional 12 h to allow binding. Beads were washed three times with lysis buffer to remove nonspecifically bound proteins and processed for mass spectrometry analysis according to a standard mass spectrometry protocol. For western blotting, beads were resuspended in SDS-PAGE loading buffer to elute the proteins, which were then boiled for 5 min, separated by SDS-PAGE, and subjected to western blotting with primary antibodies (TXNIP, CAST).

### Calpain kinase activity assay

Cells were harvested, washed with PBS to remove residual media and debris, and then incubated on ice for 20 min with an extraction buffer from the Abcam calpain kinase kit to release cellular proteins. Cell lysates were centrifuged and their supernatant protein concentrations were measured using the BCA kit. For the calpain kinase assay, the lysate protein concentration was adjusted to 50 µg and added to a 96-well black/clear bottom plate with reaction buffer and calpain substrate. To measure calpain kinase activity, the reaction mixture was incubated for 60 min at room temperature, and fluorescence intensity was measured using a microplate reader with 500/505 nm excitation/emission wavelengths.

### Real-time quantitative RT-PCR

Total RNA was extracted from BC cell lines using the RNeasy mini kit (QIAgen). RNA concentration was determined using a nanodrop spectrophotometer. Subsequently, complementary DNA (cDNA) was synthesized from the extracted RNA using an RNA-to-cDNA kit (Takara Bio). For quantitative PCR (qPCR) analysis, we used primer sequences specific to the genes of interest and SYBR Green (QIAgen) as the detection method (Supplementary Table 1). GAPDH was selected as the internal control for gene expression normalization.

### Enzyme-Linked Immunosorbent Assay

IL-24 expression levels in the supernatants from MDA-MB-231 and HCC-1954 BC cell lines were quantified using an enzyme-linked immunosorbent assay **(**ELISA) kit according to the manufacturer’s instructions (Ray Biotech). Briefly, 100 µL supernatant was added to the appropriate wells and incubated for 2.5 h at room temperature with gentle shaking, followed by four washes. 100 µL 1X prepared biotinylated antibody was then added and the solution incubated for 1 h with gentle shaking. After a further washing, 100 µL prepared streptavidin solution was added and also washed. Next, 100 µL 3,3’,5,5’-tetramethylbenzidine (TMB) one-step substrate reagent was added and incubated for 30 min. Finally, a stop solution was added and the reading was recorded at 450 nm.

### Statistical analyses

Experiments were conducted with three or more replicates. Data are presented as mean ± standard deviation as specified in the figure legends. Statistical significance was assessed using various methods: unpaired, two-tailed Student’s *t* tests for comparisons between two groups, one-way ANOVA followed by Tukey’s multiple comparisons test for comparisons among more than two groups at a single time point, two-way ANOVA with Tukey’s multiple comparisons test for comparisons among more than two groups at multiple time points, and the Kaplan–Meier method for survival analyses. These analyses were performed using Graph Pad Prism software version 10.2. A value of *p* < 0.05 was considered statistically significant.

## Results

### Impact of TXNIP on cell proliferation, wound healing, colony formation, and tumor growth in MDA-MB-231 and HCC-1954 BC cells

**Fig. 1:**
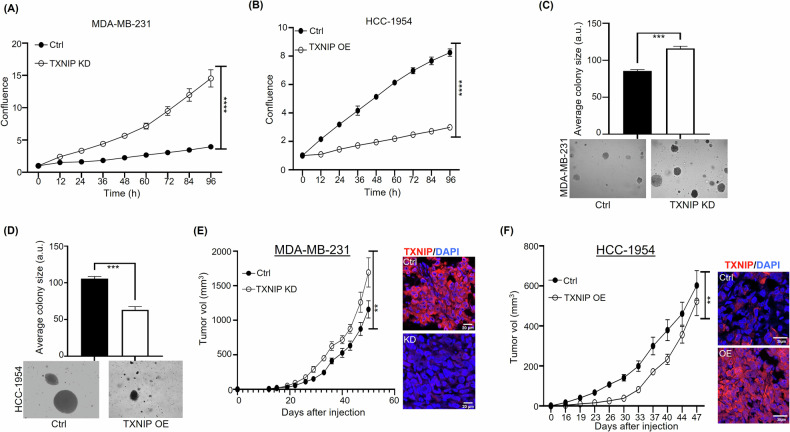
Effects of knocking down and overexpressing TXNIP on breast cancer cell proliferation, anchorage-independent soft agar assay, and tumor growth. **A**, **B** Cell proliferation over time, quantified using an IncuCyte automated live-cell analysis system, for (**A**) TXNIP-KD MDA-MB-231 cells and (**B**) TXNIP-OE HCC-1954 cells. **C**, **D** Anchorage-independent soft agar assay was performed on (**C**) TXNIP-KD MDA-MB-231 cells and (**D**) TXNIP-OE HCC-1954 cells. Representative images were quantified to determine the average colony size. **E** TXNIP knockdown in MDA-MB-231 cells, confirmed by immunofluorescence staining (*right*), promotes tumor growth (*left*). **F** TXNIP overexpression in HCC-1954 cells, confirmed by immunofluorescence staining (*right*), initially inhibits tumor growth (*left*). However, approximately 30 days after cell injection, TXNIP-OE HCC-1954 tumors display accelerated growth relative to wild-type HCC-1954 tumors. *p < 0.05, **p < 0.01, ***p < 0.001. ****p < 0.0001 versus control.

Based on our previous study showing that TXNIP activation by UNC0642 suppressed cell proliferation and tumor growth [18], we conducted TXNIP knockdown or overexpression in BC cells to investigate the direct impact of TXNIP on BC cell growth and survival. We performed shRNA-mediated TXNIP knockdown in MDA-MB-231 cells, which have a high level of endogenous TXNIP expression (Supplementary Fig. 1A). Conversely, we overexpressed TXNIP in HCC-1954 cells, which have a low level of TXNIP expression (Supplementary Fig. 1B). Following TXNIP knockdown or overexpression, we quantified MDA-MB-231 and HCC-1954 cell proliferation over time using an IncuCyte automated live-cell analysis system. TXNIP knockdown in MDA-MB-231 cells increased cell proliferation, whereas TXNIP overexpression in HCC-1954 cells reduced proliferation (Fig. 1A, B). Wound healing assays showed that TXNIP knockdown in MDA-MB-231 cells led to faster wound closure than in WT MDA-MB-231 cells. Conversely, TXNIP-overexpression in HCC-1954 cells resulted in slower wound closure than in WT HCC-1954 cells (Supplementary Fig. 1C, D). We also used soft agar assays to assess the effects of TXNIP manipulation on the anchorage-independent growth of TXNIP-KD MDA-MB-231 cells and TXNIP-OE HCC-1954 cells. The average size of TXNIP-KD MDA-MB-231 colonies was approximately 1.36x larger than that of WT MDA-MB-231 colonies (*p* < 0.001), whereas the average size of TXNIP-OE HCC-1954 colonies was approximately 0.6x smaller than that of WT HCC-1954 colonies (*p* < 0.001) (Fig. 1C, D).

To determine the effect of TXNIP on tumor growth, we injected TXNIP-KD MDA-MB-231 cells, TXNIP-OE HCC-1954 cells, or the WT parental cell lines into the mammary fat pads of NSG mice. TXNIP knockdown significantly increased MDA-MB-231 tumor growth (Fig. 1E). In contrast, TXNIP overexpression reduced HCC-1954 tumor growth for up to 33 days following cell injection, followed by a late acceleration in tumor growth due to an unknown mechanism(s) (Fig. 1F). IF staining of dissected tumors confirmed persistent TXNIP knockdown in MDA-MB-231 tumors and TXNIP overexpression in HCC-1954 tumors (Fig. 1E, F). These results strongly support an antitumor function for TXNIP in both MDA-MB-231 cells and HCC-1954 cells.

### TXNIP overexpression in HCC-1954 cells impairs mitochondrial function and induces apoptosis

**Fig. 2:**
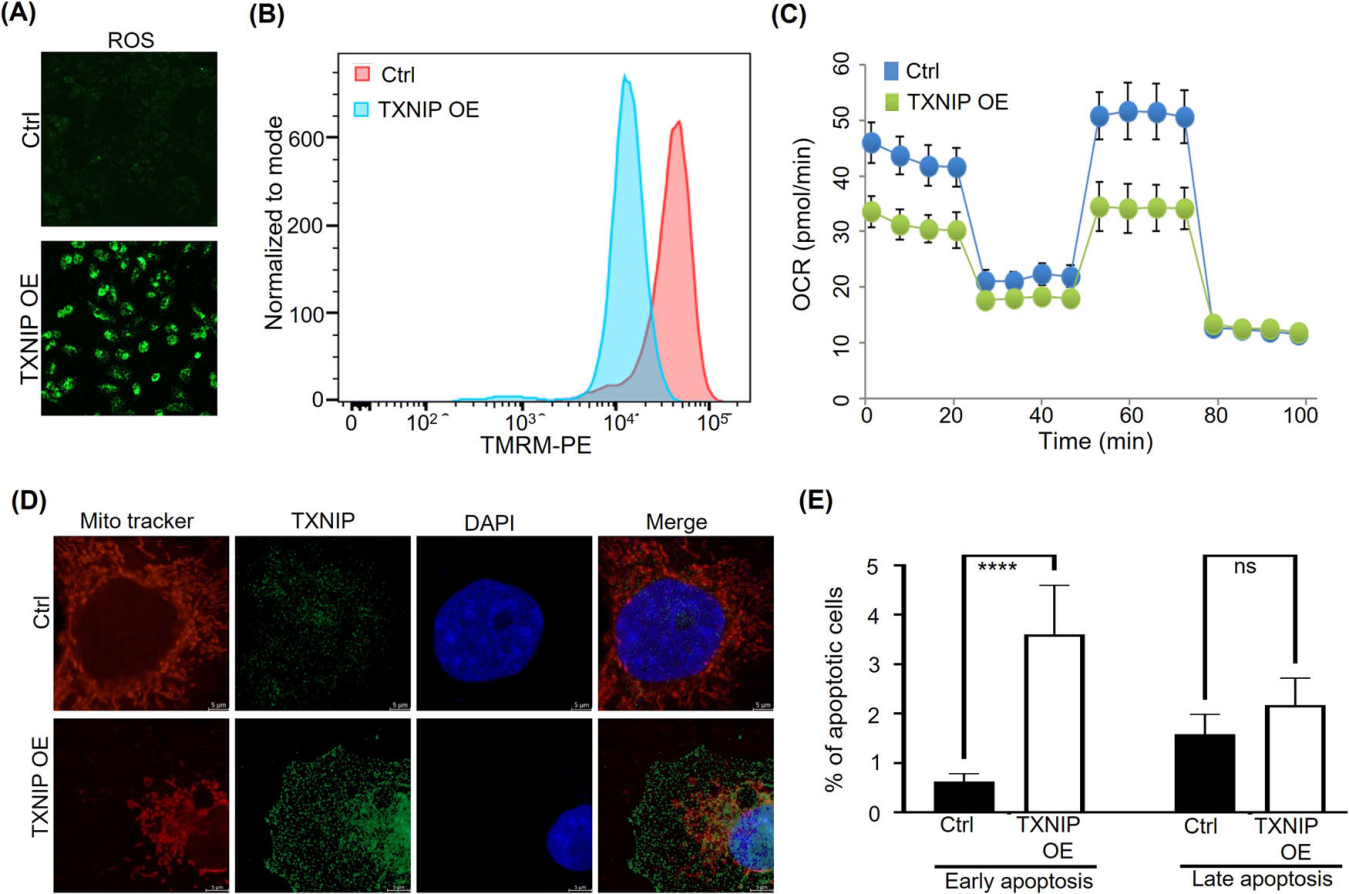
Effects of TXNIP overexpression (OE) on reactive oxygen species (ROS) levels, mitochondrial membrane potential, oxygen consumption rate, and cell viability in HCC-1954 breast cancer cells. **A** ROS levels are elevated in TXNIP-OE versus wild-type (control) HCC-1954 cells, as shown by CellROX fluorescence. **B** Mitochondrial membrane potential in TXNIP-OE and wild-type (control) HCC-1954 cells, assessed using TMRM dye. **C** The oxygen consumption rate was significantly lower in TXNIP-OE versus wild-type (control) HCC-1954 cells, as measured by the Seahorse cell mito stress assay. **D** Representative images depicting punctate mitochondria indicative in TXNIP-OE HCC-1954 versus wild-type (control) HCC-1954 cells. **E** Percentage of early apoptotic, and late apoptotic cells following TXNIP overexpression in HCC-1954 cells, measured using flow cytometry.

Previous studies show that TXNIP inhibits thioredoxin activities while increasing both ROS production and oxidative stress [23]. A CellROX assay, used to detect ROS levels, showed that TXNIP overexpression in HCC-1954 cells increases ROS production (Fig. 2A). Oxidative stress associated with ROS leads to mitochondrial DNA damage and changes in MMP [24]. To determine if TXNIP overexpression impairs mitochondrial function in HCC-1954 cells, we used the red fluorescent probe TMRM, a mitochondrial membrane potential–sensitive indicator, to detect mitochondrial membrane depolarization. TXNIP-OE HCC-1954 cells showed reduced mitochondrial membrane potential levels relative to WT HCC-1954 cells (Fig. 2B), suggesting that TXNIP negatively impacts mitochondrial membrane potential and mitochondrial function. TXNIP-OE HCC-1954 cells also exhibited a reduced oxygen consumption rate relative to WT HCC-1954 cells (Fig. 2C), confirming impaired mitochondrial function following TXNIP overexpression. Mitochondrial damage and loss of mitochondrial membrane potential often lead to mitophagy, which involves the sequestration and degradation of mitochondria by the autophagy-lysosome pathway [25]. Using a red fluorescent MitoTracker dye, a cell-permeable dye that selectively accumulates in active mitochondria [26], we showed that punctate mitochondria were more visible in TXNIP-OE HCC-1954 cells than in WT HCC-1954 cells, as indicated by the reduced fluorescence (Fig. 2D), suggesting that TXNIP overexpression induces mitochondrial damage in HCC-1954 cells. Finally, TXNIP overexpression caused a significant increase in the percentage of apoptotic cells in HCC-1954 cells, with over a 20-fold rise observed in annexin V flow cytometry assays (Fig. 2E, Supplementary Fig. 1E). However, the absolute levels of apoptosis in HCC-1954 cells were modest under both basal conditions (~0.1%) and after TXNIP overexpression (~2.3%), suggesting a moderate role for TXNIP-induced apoptosis in affecting HCC-1954 cell fate. This limited apoptotic response likely reflects presence of the p53 Y163C mutation in HCC-1954 cells [27], which impairs p53-dependent apoptotic signaling [28].

### CAST is a novel TXNIP-interacting protein in breast cancer cells

**Fig. 3:**
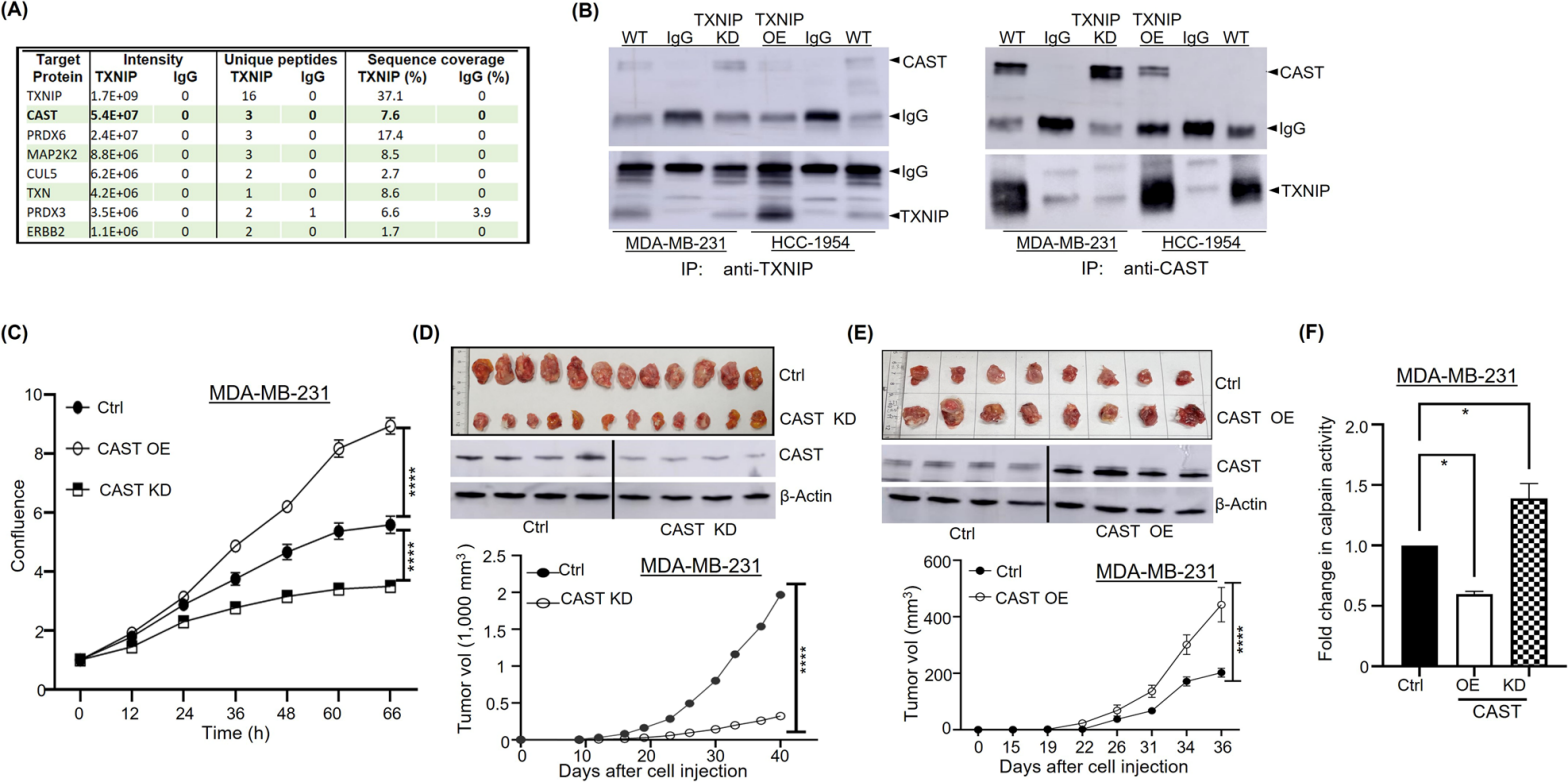
CAST is a novel TXNIP-interacting protein with tumor-promoting activity. **A** The top-ranked TXNIP-associated proteins in MDA-MB-231 and HCC-1954 cells were identified using mass spectrometry analysis and co-immunoprecipitation (co-IP) across three different experiments. This is one of the experiments. **B** The interaction between TXNIP and CAST in MDA-MB-231 cells and HCC-1954 cells was detected by co-IP, using anti-TXNIP (*left*) and anti-CAST (*right*) antibodies. **C** Cell proliferation was quantified over time using an IncuCyte automated live-cell analysis system following CAST knockdown (KD) in MDA-MB-231 cells and CAST overexpression (OE) in HCC-1954 cells. **D** Tumor growth curves (*bottom*) and images of xenograft tumors (*top*) from CAST-KD and wild-type (control) MDA-MB-231 cells following their orthotopic injection and growth in NSG mice. CAST knockdown in MDA-MB-231 tumors was confirmed by western blotting (*middle*). **E** Tumor growth curves (*bottom*) and images of xenograft tumors (*top*) from CAST-OE and wild-type (control) MDA-MB-231 cells following their orthotopic injection and growth in NSG mice. CAST overexpression in MDA-MB-231 tumors was confirmed by western blotting (*middle*). **F** CAST knockdown in MDA-MB-231 cells decreased calpain kinase activity, whereas CAST overexpression reduced calpain kinase activity. *p < 0.05, **p < 0.01, ***p < 0.001. ****p < 0.0001 versus control.

Next, we sought to elucidate the mechanism underlying TXNIP’s anticancer activity. To identify TXNIP-associated proteins that might regulate tumor growth and survival, we performed co-IP followed by mass spectrometry in TXNIP-KD MDA-MB-231 cells and TXNIP-OE HCC-1954 cells. In three independent experiments, we consistently identified CAST as a top-ranked TXNIP-associated protein (Fig. 3A). Interrogation of the STRING database, a resource for exploring protein-protein interactions, revealed SLC2A1 (GLUT-1), NEDD4L, and ITCH, but not CAST, as known interacting partners for TXNIP (Supplementary Fig. 2A). To confirm TXNIP-CAST interaction in MDA-MB-231 and HCC-1954 cells, we performed reciprocal co-IP experiments using either anti-CAST or anti-TXNIP antibodies. The results consistently confirmed the TXNIP-CAST interaction (Fig. 3B), which was also replicated in T47D BC cells (Supplementary Fig. 2B). Notably, while endogenous CAST expression is low in WT HCC-1954 cells, CAST was robustly upregulated in TXNIP-OE HCC-1954 cells, indicating a potential negative feedback mechanism in which elevated CAST levels might counteract TXNIP’s antitumor activity in TXNIP-OE HCC-1954 cells.

### CAST enhances MDA-MB-231 cell proliferation and tumor growth

Next, we investigated CAST’s function in BC cell growth and survival by generating CAST-KD MDA-MB-231 cells and CAST-OE MDA-MB-231 cells. CAST knockdown in MDA-MB-231 cells significantly reduced cell proliferation, 2D colony formation, and 3D anchorage-independent growth, whereas CAST overexpression had the opposite effects (Fig. 3C; Supplementary Fig. 3A, B). However, no discernable differences in ROS levels between the different cell types were detected (Supplementary Fig. 3C). Consistent with these in vitro results, CAST-KD MDA-MB-231 tumors in NSG mice exhibited reduced tumor volume and weight relative to WT MDA-MB-231 tumors, whereas CAST-OE MDA-MB-231 tumors displayed greater tumor volume and weight relative to WT MDA-MB-231 tumors (Fig. 3D, E; Supplementary Fig. 3D–G). We confirmed persistent CAST knockdown and overexpression in vivo in tumors from MDA-MB-231 xenografts by western blotting and IF staining of tumors recovered at necropsy (Fig. 3D, E). Taken together, these results suggest that CAST plays a tumor-promoting role in MDA-MB-231 cells.

In light of CAST’s function as an endogenous inhibitor of calpains, we also investigated the impact of CAST knockdown and overexpression on calpain kinase activity in MDA-MB-231 cells. As shown in Fig. 3F, CAST overexpression led to a 0.59-fold decrease (*p* < 0.05) in calpain kinase activity relative to WT MDA-MB-231 cells, whereas CAST knockdown resulted in a 1.38-fold increase (*p* < 0.05) in calpain kinase activity compared to WT MDA-MB-231 cells.

### CAST enhances HCC-1954 cell proliferation and tumor growth

**Fig. 4:**
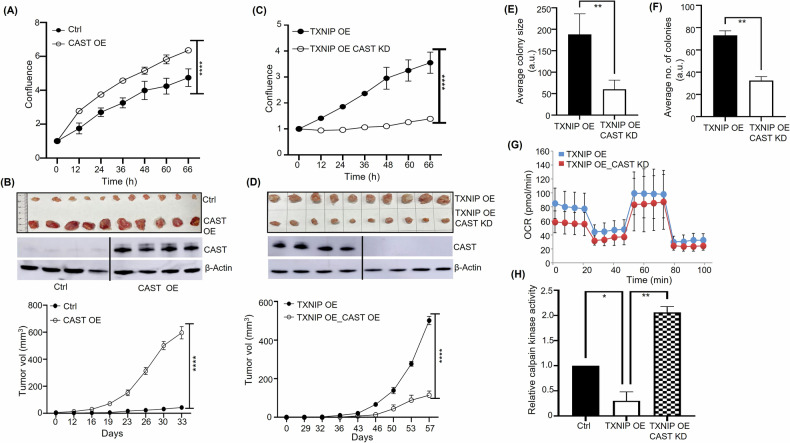
Effects of CAST overexpression (OE) and knockdown (KD) on cell proliferation, tumor growth, oxygen consumption rate, and calpain kinase activity in HCC-1954 breast cancer cells. **A** Cell proliferation, quantified using an IncuCyte automated live-cell analysis system, was greater in CAST-OE versus wild-type (control) HCC-1954 cells. **B** Tumor growth curves (*bottom*) and images of xenograft tumors (*top*) from CAST-OE versus wild-type (control) HCC-1954 cells following their orthotopic injection and growth in NSG mice. CAST overexpression in HCC-1954 tumors was confirmed by western blotting (*middle*). **C** Cell proliferation, quantified using an IncuCyte automated live-cell analysis system, was lower in CAST-KD_TXNIP-OE versus TXNIP-OE HCC-1954 cells. **D** Tumor growth curves (*bottom*) and images of xenograft tumors (*top*) from CAST-KD_TXNIP-OE and TXNIP-OE HCC-1954 cells following their orthotopic injection and growth in NSG mice. CAST knockdown in CAST-KD_TXNIP-OE HCC-1954 tumors was confirmed by western blotting (*middle)*. Soft agar assays quantified anchorage-independent growth in terms of (**E**) colony size and (**F**) colony number in CAST-KD_TXNIP-OE and TXNIP-OE HCC-1954 cells. **G** The oxygen consumption rate was significantly lower in CAST-KD_TXNIP-OE versus TXNIP-OE HCC-1954 cells, as measured by the Cell Mito Stress kit (Seahorse). **H** Calpain kinase activity was significantly lower in CAST-KD_TXNIP-OE versus TXNIP-OE HCC-1954 cells. *p < 0.05, **p < 0.01, ***p < 0.001. ****p < 0.0001 versus control.

Since CAST exhibited a tumor-promoting function in MDA-MB-231 cells, we investigated whether CAST plays a similar role in HCC-1954 cells. Unlike MDA-MB-231 cells, HCC-1954 cells have a very low level of endogenous CAST expression. Therefore, we generated an HCC-1954 cell line with CAST overexpression. When compared with WT HCC-1954 cells, CAST-OE HCC-1954 cells exhibited increased cell proliferation (Fig. 4A), colony number, and colony size (Supplementary Fig. 4A, B). Relative to WT HCC-1954 cells, CAST-OE HCC-1954 cells also demonstrated accelerated tumor growth in NSG mice, as evidenced by greater tumor volumes and weights (Fig. 4B, Supplementary Fig. 4C, D). As expected, western blotting and IF staining confirmed persistent CAST overexpression in CAST-OE HCC-1954 tumors. Taken together, these findings further establish CAST’s tumor-promoting activity. Similar to our findings in MDA-MB231 cells, no significant difference in ROS levels was detected between CAST-OE HCC-1954 cells and WT HCC-1954 cells (Supplementary Fig. 4E), suggesting that CAST’s tumor-promoting activity might function independent of ROS signaling.

### CAST knockdown in TXNIP-overexpressing HCC-1954 cells reduces cell proliferation and tumor growth

Since CAST has tumor-promoting activity, we hypothesized that CAST upregulation in TXNIP-OE HCC-1954 cells (Supplementary Fig. 5A) might explain the late-onset acceleration in the growth of TXNIP-OE HCC-1954 tumors in NSG mice (Fig. 1F). To test this hypothesis, we KD CAST expression in TXNIP-OE HCC-1954 cells. As predicted, cell proliferation was diminished in CAST-KD_TXNIP-OE HCC-1954 versus TXNIP-OE HCC-1954 cells (Fig. 4C). Relative to TXNIP-OE HCC-1954 cells, CAST-KD_TXNIP-OE HCC-1954 cells also displayed attenuated colony growth and proliferation (Fig. 4E, F**;** Supplementary Fig. 5B, C). Following transplantation into NSG mice, the volume and weight of CAST-KD_TXNIP-OE HCC-1954 tumors were lower than that of TXNIP-OE HCC-1954 tumors (Fig. 4D**;** Supplementary Fig. 5D). Notably, while mice harboring TXNIP-OE HCC-1954 cells tend to die prematurely without significant tumor burden or visible metastasis, all mice harboring CAST-KD_TXNIP-OE HCC-1954 cells survived for the entire experiment (Supplementary Fig. 5E), suggesting that CAST depletion could diminish the lethality caused by TXNIP-OE HCC-1954 tumors.

To assess whether CAST’s effect on mitochondrial function could help explain its impact on HCC-1954 cell proliferation and survival rates, we measured changes in oxygen consumption rate (OCR)–related metabolic features using the Seahorse cell mito stress assay. Basal OCR decreased from 47% in TXNIP-OE HCC-1954 cells to 32% in CAST-KD_TXNIP-OE HCC-1954 cells, concurrent with a decrease in ATP production from 32% in CAST-KD_TXNIP-OE cells to 23% in CAST-KD_TXNIP-OE HCC-1954 cells (Fig. 4G**;** Supplementary Fig. 5F). While these changes appear modest, they consistently indicate a measurable impact of CAST knockdown on the metabolic fitness of HCC-1954 cells, which warrants further investigation. Together, these results could also partly explain the tumor-promoting function of CAST, which was upregulated in TXNIP-OE HCC-1954 cells, by counteracting the tumor suppressor activity of TXNIP. Consistent with CAST’s role as a calpain inhibitor, we confirmed that calpain kinase activity was greater in CAST-KD_TXNIP-OE HCC-1954 cells than in TXNIP-OE HCC-1954 cells (Fig. 4H). It remains unclear whether enhanced calpain kinase activity contributed directly to reduced CAST-KD_TXNIP-OE HCC-1954 cell proliferation and tumor growth.

### CAST knockdown in TXNIP-overexpressing HCC-1954 cells induces G1 phase cell cycle arrest

**Fig. 5:**
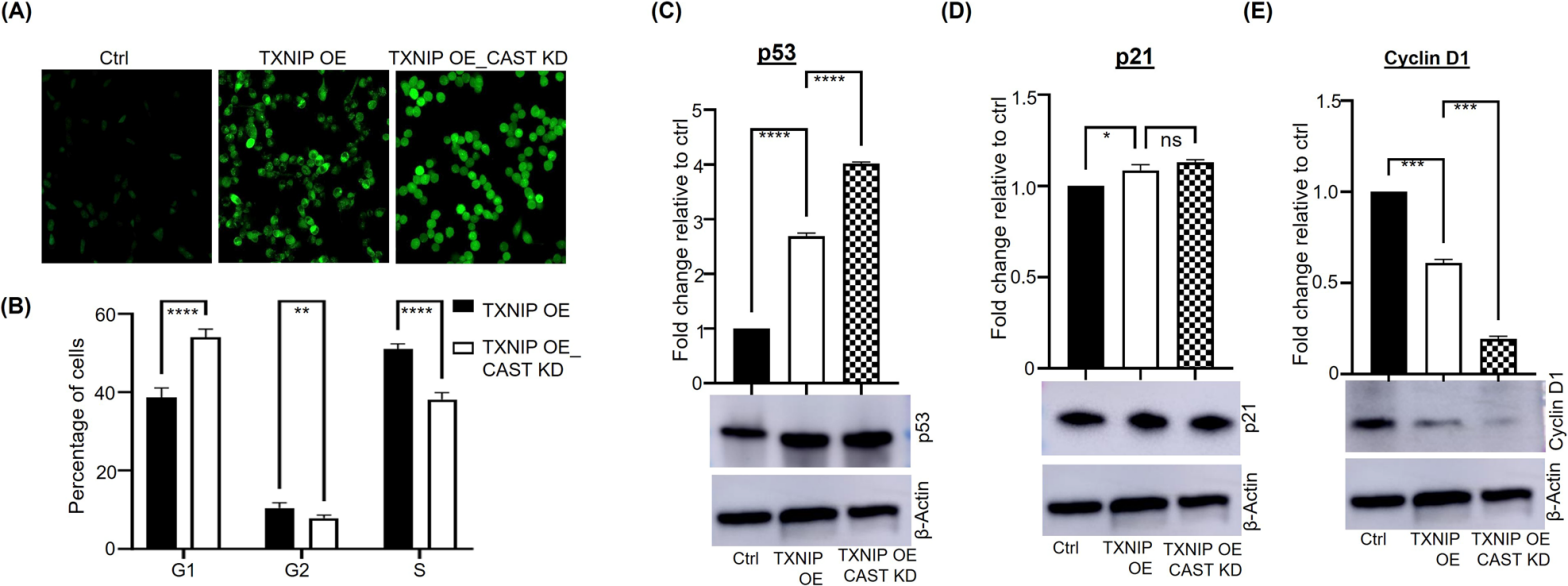
CAST knockdown (KD) in TXNIP-overexpressing (OE) HCC-1954 cells increases apoptosis and cell cycle arrest at the G1 phase. **A** Reactive oxygen species levels were significantly higher in CAST-KD_TXNIP-OE versus TXNIP-OE and wild-type (control) HCC-1954 cells. **B** Histograms illustrating altered distributions of cells in G1, G2, and S phases in CAST-KD_TXNIP-OE versus TXNIP-OE HCC-1954 cells. Expression of (**C**) p53, (**D**) p21, and (**E**) cyclin-D1 in CAST-KD_TXNIP-OE versus TXNIP-OE HCC-1954 cells as measured by western blotting, using β-actin as a loading control. Results presented as fold change relative to wild-type (control) cells, based on means ± SD from three experiments. *p < 0.05, **p < 0.01, ***p < 0.001. ****p < 0.0001 versus control.

CAST-KD_TXNIP-OE HCC-1954 cells displayed a robust increase in ROS production relative to TXNIP-OE HCC-1954 cells (Fig. 5A); TXNIP-OE HCC-1954 cells also showed increased ROS production relative to WT HCC-1954 cells (Fig. 2A). We hypothesized that depleting CAST in TXNIP-OE HCC-1954 cells might release CAST’s inhibitory effect on TXNIP activity, increasing ROS levels and subsequently inhibiting cell proliferation. As predicted, relative to TXNIP-OE HCC-1954 cells, CAST-KD_TXNIP-OE HCC-1954 cells were significantly more likely to be arrested in the G1 phase (57.92% versus 35.74%, *p* < 0.0001) and less likely to enter S phase (35.33% versus 51.34%, *p* < 0.0001) (Fig. 5B; Supplementary Fig. 5G, H). CAST knockdown–induced cell cycle arrest was associated with an upregulation of p53, but not p21, and with a downregulation of cyclin D1 (Fig. 5C–E). HCC-1954 cells express the p53 Y163C mutation [27] that abolishes p53 transcriptional activity and drastically reduces its ability to inhibit cell growth [28]. The absence of p21 upregulation reflects the inability of p53 Y163C to regulate transcription [28]. CAST knockdown-induced G1 cell cycle arrest is more likely mediated primarily by its ability to reduce cyclin D1 expression (Fig. 5E) [29]. These findings reveal for the first time that CAST knockdown leads to G1 phase cell cycle arrest, accompanied by dysregulation of cyclin D1, a critical cell cycle regulator, in response to TXNIP overexpression in HCC-1954 cells.

### TXNIP expression leads to IL-24 activation

To further understand the mechanism underlying TXNIP’s anticancer activity in BC cells, we sought to identify TXNIP’s downstream targets in MDA-MB-231 and HCC-1954 cells. Thus, we performed RNA-seq-based gene expression profiling to discern genes differentially expressed between TXNIP-KD MDA-MB-231 cells and WT MDA-MB-231 cells, and between TXNIP-OE HCC-1954 cells and WT HCC-1954 cells. Among the many differentially expressed genes identified, IL-24 expression was downregulated in TXNIP-KD versus WT MDA-MB-231 cells but upregulated in TXNIP-OE versus WT HCC-1954 cells (Supplementary Table 2), suggesting a positive association between TXNIP and IL-24 expression. Several studies have reported that IL-24 triggers apoptosis in cancer cells, including MDA-MB-231 BC cells, without killing normal cells [30, 31], making it an ideal target for cancer therapy. In addition to TXNIP upregulation [18], UNC0642 treatment also upregulated IL-24 expression in MDA-MB-231 cells (Supplementary Table 2), further supporting a positive association between TXNIP and IL-24 activity.

**Fig. 6:**
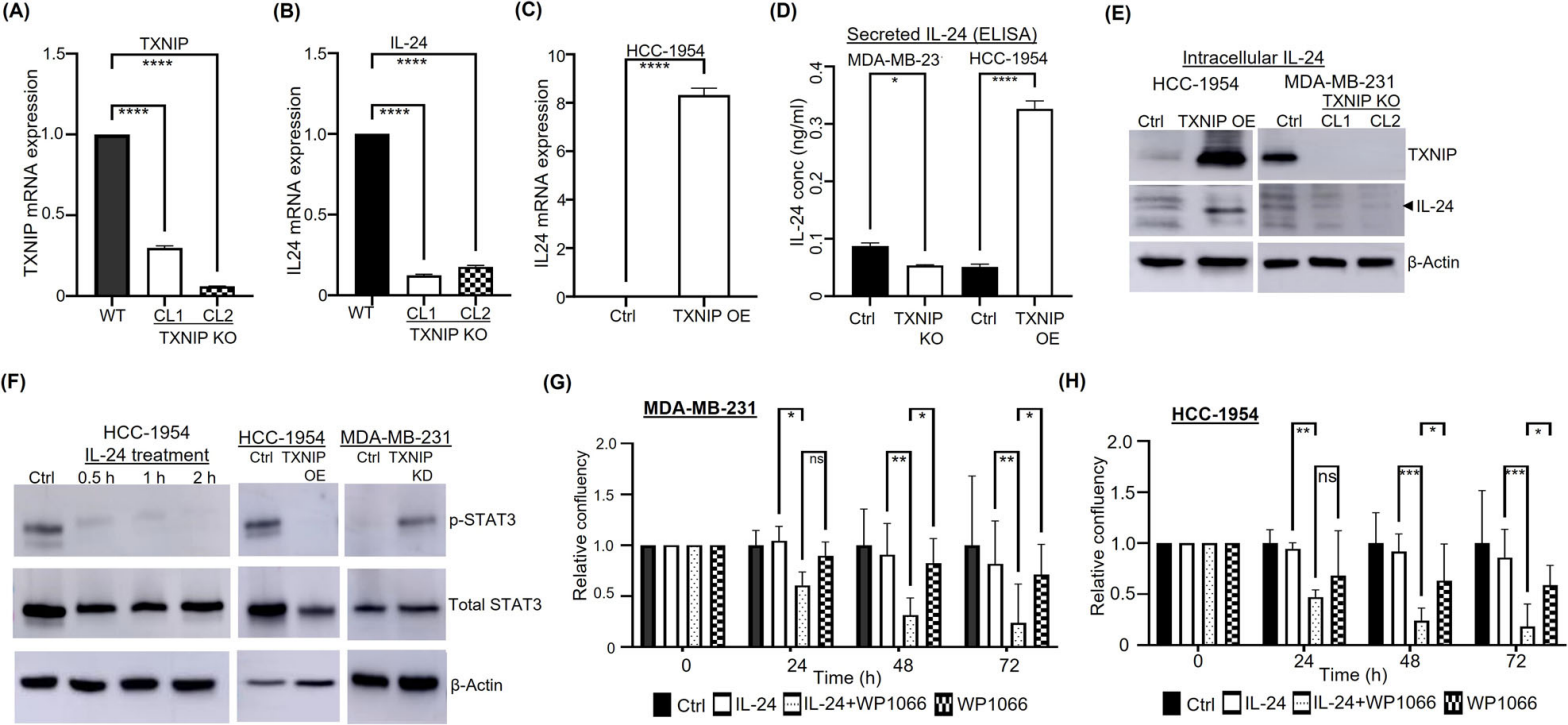
Crosstalk between TXNIP, IL-24, and STAT3 signaling in breast cancer cells. **A**–**C** qRT-PCR analysis revealed that (**A**) TXNIP knockout (KO) in MDA-MB-231 cells (**B**) decreases IL-24 expression (CL1 and CL2 denote two distinct TXNIP-KO MDA-MB-231 clones). **C** Conversely, TXNIP overexpression (OE) in HCC-1954 cells significantly increases IL-24 expression. **D**, **E** IL-24 protein expression is lower in TXNIP-KO versus wild-type (control) MDA-MB-231 cells, and higher in TXNIP-OE versus wild-type (control) HCC-1954 cells, both (**D**) extracellularly, as determined by ELISA, and (**E**) intracellularly, as confirmed by western blotting. **F** p-STAT3 and total STAT3 levels in: HCC-1954 cells treated with 100 ng/ml concentrations of rhIL-24 for different time durations (*left*); TXNIP-OE, CAST-OE, CAST-KD_TXNIP-OE, and wild-type (control) HCC-1954 cells (*middle*); and TXNIP-KD, CAST-OE, and wild-type (control) MDA-MB-231 cells (*right*). Relative to treatment with IL-24 or WP1066 alone, treatment with IL-24 plus WP1066 caused greater inhibition of cell proliferation in (**G**) MDA-MB-231 and (**H**) HCC-1954 cells. *p < 0.05, **p < 0.01, ***: *p* < 0.001. ****p < 0.0001 versus control.

Since no prior study had established a link between TXNIP and IL-24 in cancer development, we conducted further validation and functional assays of IL-24 due to its recognized role in cancer-specific cytotoxicity. To validate the RNA-seq results, we performed qRT-PCR to quantify IL-24 expression in TXNIP-KD and TXNIP-KO MDA-MB-231 cells, TXNIP-OE HCC-1954 cells, and the respective WT cell lines. qRT-PCR results confirmed that depleting TXNIP expression significantly reduced IL-24 expression in TXNIP-KD and TXNIP-KO MDA-MB-231 cells (Fig. 6A, B; Supplementary Fig. 5I). In contrast, IL-24 expression was significantly higher in TXNIP-OE versus WT HCC-1954 cells (Fig. 6C), corroborating the positive association between TXNIP and IL-24 expression. IL-24 upregulation by TXNIP was further validated at the protein level, both intracellularly through extracellularly via ELISA and western blotting (Fig. 6D, E).

### TXNIP expression and IL-24 treatment reduces STAT3 phosphorylation

Next, we asked whether IL-24 affects TXNIP expression by treating HCC-1954 cells with recombinant human IL-24 (rhIL-24). Western blotting results showed that rhIL-24 had no detectable effect on TXNIP expression (Supplementary Fig. 5J). However, rhIL-24 treatment significantly attenuated phosphorylated STAT3 levels in HCC-1954 cells (Fig. 6F), suggesting IL-24’s anticancer activity, at least in part, may result from dampening the STAT3 signaling axis. Furthermore, in TXNIP-low HCC-1954 cells, p-STAT3 was easily detectable. Upon TXNIP OE, p-STAT3 was reduced significantly. In TXNIP-high MDA-MB231 cells, p-STAT3 was minimally detectable but increased substantially upon TXNIP knockdown (Fig. 6F). These findings indicate a negative regulation of STAT3 signaling by TXNIP and IL-24 in BC cells.

WP1066, a selective STAT3 inhibitor, can inhibit MDA-MB-231 tumor growth and brain metastasis in a mouse BC model [32]. Both IL-24 and WP1066 have been studied as cancer therapies in clinical trials [33, 34]. Because of the novel crosstalk we identified here between TXNIP, IL-24, and STAT3, we investigated the therapeutic potential of co-targeting the TXNIP–IL-24–STAT3 axis. As expected, WP1066 treatment markedly reduced p-STAT3 levels in HCC-1954 cells (Supplementary Fig. 5K). Next, we subjected MDA-MB-231 and HCC-1954 cells to IL-24 and WP1066 treatment either alone or in combination. IL-24 treatment did not exert any discernible impact on cell viability, but WP1066 treatment markedly inhibited the proliferation of both MDA-MB-231 and HCC-1954 cells (Fig. 6G, H). Combined IL-24 and WP1066 treatment elicited more pronounced growth suppression in both MDA-MB-231 and HCC-1954 cells than WP1066 treatment alone (Fig. 6G, H), suggesting that IL-24 may sensitize breast cancer cells to WP1066. Finally, relative to WT HCC-1954 cells, TXNIP-OE HCC-1954 cells displayed increased sensitivity to IL-24 and WP1066 treatment (Supplementary Fig. 5L). Further studies are needed to elucidate the role of IL-24 in mediating TXNIP’s antitumor activity.

## Discussion

In this study, we have demonstrated TXNIP’s anticancer effects in MDA-MB-231 (high endogenous TXNIP) and HCC-1954 (low endogenous TXNIP) breast cancer cells. Our investigation aimed to uncover the mechanisms behind TXNIP’s tumor-suppressive actions to inform the development of new therapeutic strategies. While we did not detect TXNIP partners such as GLUT-1, NEDD4L, and ITCH as reported previously in other cell types [2, 35–37], it remains to be determined whether the TXNIP-CAST interaction is broadly conserved across other tissues and cell types. Additionally, no study has examined whether post-translational modifications of TXNIP or CAST might modulate their interaction. Further research is needed to define the broader function of the TXNIP-CAST interaction in development and to identify key regulators of their interaction in cancer cells.

Functional studies revealed that CAST exhibits tumor-promoting activity in both MDA-MB-231 and HCC-1954 cells. CAST is an endogenous inhibitor of calpains (m-calpain and μ-calpain) [38] but its involvement in cancer biology remains poorly understood. In gastric cancer, elevated CAST expression is significantly linked to reduced overall survival [39], suggesting its tumor-promoting potential. Conversely, in breast cancer patients, CAST mRNA and protein levels show an inverse relationship with the poor prognostic indicator of lymphovascular invasion [40], indicating possible anti-tumor activity. These findings highlight the complex, context-dependent roles of CAST in cancer, underscoring the necessity for further research to elucidate its dual functions and potential as a therapeutic target.

Initially, TXNIP overexpression in HCC-1954 cells inhibited tumor growth as expected. However, these cells later showed an unexpected acceleration in tumor growth after 4 weeks; the mechanism(s) for this observation remains unclear (Fig. 1F). WT HCC-1954 cells have minimal detectable levels of TXNIP and CAST. Overexpressing TXNIP in this cell line significantly increased CAST expression. Given CAST’s role as a tumor promoter, the elevated CAST levels in TXNIP-OE HCC-1954 cells may counteract TXNIP’s antitumor effects. This outcome could explain the observed pattern of initial tumor growth inhibition, where the subsequent accelerated growth after four weeks may reflect an acquired resistance to this inhibition. Consistent with the initial inhibitory events, knocking down CAST in TXNIP-OE HCC-1954 cells led to a significant reduction in cell proliferation, colony formation, and tumor growth in a mouse xenograft model (Fig. 4C–F). These findings suggest that CAST modulates TXNIP’s tumor-suppressive activity in breast cancer cells, highlighting its potential as a target for therapeutic intervention.

In addition to identifying the novel interaction between CAST and TXNIP, our study revealed that overexpressing TXNIP increases IL-24 levels while knocking down TXNIP decreases IL-24 expression in breast cancer cells. This is the first demonstration that TXNIP positively affects IL-24 expression in breast cancer cells, although the mechanism underlying this positive correlation is unclear. Several studies have reported that IL-24 can be activated by transcription factors such as AP-1, STAT6, and NF-κB [41–44]. TXNIP, while not directly regulating gene expression, inhibits thioredoxin, leading to increased ROS production (Fig. 2A). Elevated ROS further enhances the activity of AP-1, STAT6, and NF-κB to increase IL-24 expression [45–47]. Additionally, ROS can also promote the translation of IL-24 translation [48], serving as another pathway through which TXNIP might enhance IL-24 expression. IL-24 can induce apoptosis in cancer cells, including MDA-MB-231 cells, without affecting normal cells [49–53], an attractive feature for a potential therapeutic target. IL24’s efficacy has been demonstrated in Phase I clinical trials for several advanced cancers [54, 55]. While the precise mechanisms behind its cancer cell-specific cytotoxicity are still under investigation, our findings suggest that TXNIP might inhibit cancer cell growth and survival by activating IL-24 signaling. These insights highlight the potential of targeting the TXNIP-IL-24 pathway as a therapeutic strategy in breast cancer, opening up a potential avenue for future research and treatment development.

IL-24 is a member of the IL-10 cytokine family, signaling via the JAK/STAT3 signaling pathway to induce cancer cell apoptosis [56, 57]. While TXNIP and IL-24 act as tumor suppressors, STAT3 is considered an oncogene, being constitutively activated in nearly 70% of solid and hematological tumors [58–61]. In addition to the inverse relationship between TXNIP expression and STAT3 phosphorylation we find in BC cells, we further show that recombinant IL-24 treatment reduces STAT3 phosphorylation, supporting IL-24’s role in inhibiting STAT3 activation [62]. However, the functional relationship between IL-24 and STAT3 signaling is complex and remains controversial. In human keratinocytes and intestinal epithelial cells, the binding of IL-24 to its receptors leads to STAT3 activation [63, 64]. Conversely, in human leukemia cells, IL-24 induces apoptosis by dephosphorylating (deactivating) STAT3 and stabilizing p53 expression [65]. This discrepancy highlights our limited understanding of IL-24’s diverse effects on different cell types through various signaling pathways. Furthermore, whether TXNIP suppresses STAT3 signaling through IL-24 activation remains to be determined in future studies.STAT3 is an established therapeutic target due to its role in promoting tumorigenesis and its association with chemotherapy resistance in aggressive tumors such as TNBC and HER2 + BC [66, 67]. In normal tissues, STAT3 activation is transient and tightly regulated by signaling molecules such as JAKs [68]. Both IL-24 and WP1066, a specific STAT3 inhibitor, have been investigated as cancer therapies in clinical trials [33, 34]. Our results show that WP1066 treatment suppressed cell growth concurrent with reduced p-STAT3 levels. Notably, combined IL-24 and WP1066 treatment caused significantly more growth suppression in both MDA-MB-231 and HCC-1954 cells than each single treatment did, highlighting the therapeutic potential of co-targeting IL-24 and STAT3 in inhibiting BC cell growth. Furthermore, we found that cells with high TXNIP expression were more sensitive to IL-24 and WP1066, suggesting TXNIP expression’s potential use as a prognostic marker for treatment response.

We acknowledge some limitations in the present study. First, while the TXNIP-CAST interaction may have relevance in breast cancer, further studies are required to explore its broader relevance, tissue-specific regulation, and any role of post-translational modifications in the interaction. Second, the unexpected acceleration in tumor growth observed in TXNIP-overexpressing HCC-1954 cells after four weeks suggests that additional, unknown mechanism(s) may influence TXNIP’s anticancer effects. Third, although our findings indicate that TXNIP may suppress cancer cell growth through IL-24 signaling, the exact mechanisms remain unclear and require further investigation. Lastly, the complex roles of CAST and IL-24 in cancer underscore the need for more comprehensive studies to fully understand the diverse effects and therapeutic potential of these molecules. While beyond the scope of the current study, investigations into these additional aspects of TXNIP, CAST, and IL-24 are ongoing.

In conclusion, our study demonstrates TXNIP’s multifaceted role in breast cancer, influencing redox balance, mitochondrial function, and cell fate. The interaction between CAST and TXNIP might diminish TXNIP’s antitumor activity. Conversely, TXNIP could suppress tumor growth by upregulating IL-24 and downregulating p-STAT3 signaling. These findings underscore TXNIP’s central role in an intricate regulatory network and its potential as a therapeutic target in cancer treatment. Further research into the interactions between TXNIP, CAST, and IL-24 is essential to better understand their roles in cancer. Exploring these interactions in patient samples could reveal valuable prognostic markers or therapeutic targets, enhancing our understanding of TXNIP and its targets in cancer pathogenesis and treatment response.

## Supplementary information

Supplementary Figure Legends

Supplementary Figure 1

Supplementary Figure 2

Supplementary Figure 3

Supplementary Figure 4

Supplementary Figure 5-1

Supplementary Figure 5-2

Supplementary Table

Orginal Western Blots

## Data Availability

The data generated during the current study are available from the corresponding author upon reasonable request.
