# Supplementary material for: Molecular mechanisms underlying TXNIP’s anti-tumor role in breast cancer, including interaction with a novel, pro-tumor partner: CAST
Source: Cell Death Dis. 2025 Apr 2;16(1):236. doi: 10.1038/s41419-025-07566-4 (PMC11965567; doi:10.1038/s41419-025-07566-4)
Supplement: Supplementary file 1 — Supplementary Figure Legends [file 41419_2025_7566_MOESM1_ESM.docx]

**Supplementary Figure Legends**

**Fig 1. Impact of TXNIP knockdown (KD) and overexpression (OE) on wound healing, in MDA-MB-231 and HCC-1954 breast cancer cells.**

**(A,B**) Western blotting and immunofluorescence staining confirm (**A**) TXNIP knockdown (KD) in MDA-MB-231 cells and (**B**) TXNIP overexpression (OE) in HCC-1954 cells. Since data are shown in separate panels for clarity, both cell line extracts were loaded on the same gel to ensure a valid comparison of TXNIP levels.

(**C,D**) Wound healing assays conducted on (**C**) TXNIP-KD MDA-MB-231 cells and (**D**) TXNIP-OE HCC-1954 cells. (**E**) Scatterplots illustrating the percentage of apoptotic cells in WT (control) HCC-1954 cells (*left*) and TXNIP-OE HCC-1954 cells (*right*). *p<0.05, **p<0.01, ***: p<0.001. ****p<0.0001 versus control.

**Fig 2. Analysis of TXNIP-interacting proteins using the STRING database and confirmation of TXNIP-CAST interaction using co-immunoprecipitation (IP).**

(**A**) TXNIP-interacting proteins identified in the STRING database.

(**B**) co-IP using antibodies against TXNIP and CAST in TXNIP-overexpressing (OE) and TXNIP-knockdown (KD) T47D breast cancer cells.

**Fig 3. Impact of CAST knockdown (KD) and overexpression (OE) on colony formation, reactive oxygen species production, and tumor growth in MDA-MB-231 breast cancer cells.**

(**A**) Soft agar assay data showing that the number of colonies was higher in CAST-OE versus wild-type (control) MDA-MB-231 cells and lower in CAST-KD versus wild-type (control) MDA-MB-231 cells.

(**B**) Soft agar assay data showing that the size of colonies was larger in CAST-OE versus wild-type (control) MDA-MB-231 cells and smaller in CAST-KD versus wild-type (control) MDA-MB-231 cells.

(**C**) Reactive oxygen species levels in CAST-KD, CAST-OE, and wild-type (control) MDA-MB-231 cells, as assessed using the CellROX dye.

(**D**) CAST expression in CAST-KD and wild-type (control) MDA-MB-231 tumor samples, determined by immunofluorescence.

(**E**) Average weight of CAST-KD and wild-type (control) MDA-MB-231 tumors.

(**F**) CAST expression in CAST-OE and wild-type (control) MDA-MB-231 tumor samples, determined by immunofluorescence. (**G**) The average weight of CAST-OE and wild-type (control) MDA-MB-231 tumors. *p<0.05, **p<0.01, ***: p<0.001. ****p<0.0001 versus control.

**Fig 4. Impact of CAST overexpression (OE) on anchorage-independent growth, colony formation, tumor growth, and reactive oxygen species levels in HCC-1954 cells.**

(**A**) Representative images of a soft agar assay evaluating anchorage-independent growth in CAST-OE and wild-type (control) HCC-1954 cells.

(**B**) Representative images of a colony formation assay in CAST-OE and wild-type (control) HCC-1954 cells.

(**C**) CAST expression in CAST-OE and wild-type (control) HCC-1954 cells, as determined by immunofluorescence.

(**D**) Average weight of CAST-OE and wild-type (control) HCC-1954 tumors.

(**E**) Reactive oxygen species levels in CAST-OE and wild-type (control) HCC-1954 cells, were assessed using CellROX dye. *p<0.05, **p<0.01, ***: p<0.001. ****p<0.0001 versus control.

**Fig 5. Impact of CAST knockdown (KD) on anchorage-independent growth, cell cycle progression, and tumor growth in TXNIP-overexpressing (OE) HCC-1954 breast cancer cells.**

(**A**) CAST and TXNIP expression in TXNIP-OE and wild-type (control) HCC-1954 cells.

(**B**) Representative images of soft agar assay evaluating anchorage-independent growth in CAST-KD_TXNIP-OE and TXNIP-OE HCC-1954 cells.

(**C**) Representative images of colony formation assay in CAST-KD_TXNIP-OE and TXNIP-OE HCC-1954 cells.

(**D**) Average weight of CAST-KD_TXNIP-OE and TXNIP-OE HCC-1954 tumors

(**E**) Survival curve for NSG mice injected with CAST-KD_TXNIP-OE or TXNIP-OE HCC-1954 cells.

**(F)** Changes in ATP production and basal oxygen consumption rate (OCR) in CAST-KD_TXNIP-OE cells compared to TXNIP-OE HCC-1954 cells, as determined using the Seahorse Mito Stress test kit.

**(G,H)** Cell cycle analysis in (**G**) TXNIP-OE HCC-1954 cells and (**H**) CAST-KD_TXNIP-OE HCC-1954 cells.

(**I**) IL-24 mRNA expression in TXNIP-KD MDA-MB-231 cells and WT (control) cells.

(**J**) TXNIP expression in HCC-1954 cells treated with rhIL-24 (100ng/ml) at varying time points, measured by western blotting.

(**K**) p-STAT3 and total STAT3 expression in HCC-1954 cells treated with different concentrations (1µM, 2µM, and 4µM) of WP1066.

(**L**) Cell proliferation in MDA-MB-231 and HCC-1954 cells treated with IL-24 and WP1066. IL-24 treatment inhibits growth significantly more in TXNIP-OE versus wild-type (control) HCC-1954 cells. WP1066 treatment inhibits growth significantly more in TXNIP-OE versus wild-type (control) HCC-1954 cells. *p<0.05, **p<0.01, ***: p<0.001. ****p<0.0001 versus control. ns: not significant.
