# Supplementary figures and images for: Molecular mechanisms underlying TXNIP’s anti-tumor role in breast cancer, including interaction with a novel, pro-tumor partner: CAST
Source: Cell Death Dis. 2025 Apr 2;16(1):236. doi: 10.1038/s41419-025-07566-4 (PMC11965567; doi:10.1038/s41419-025-07566-4)

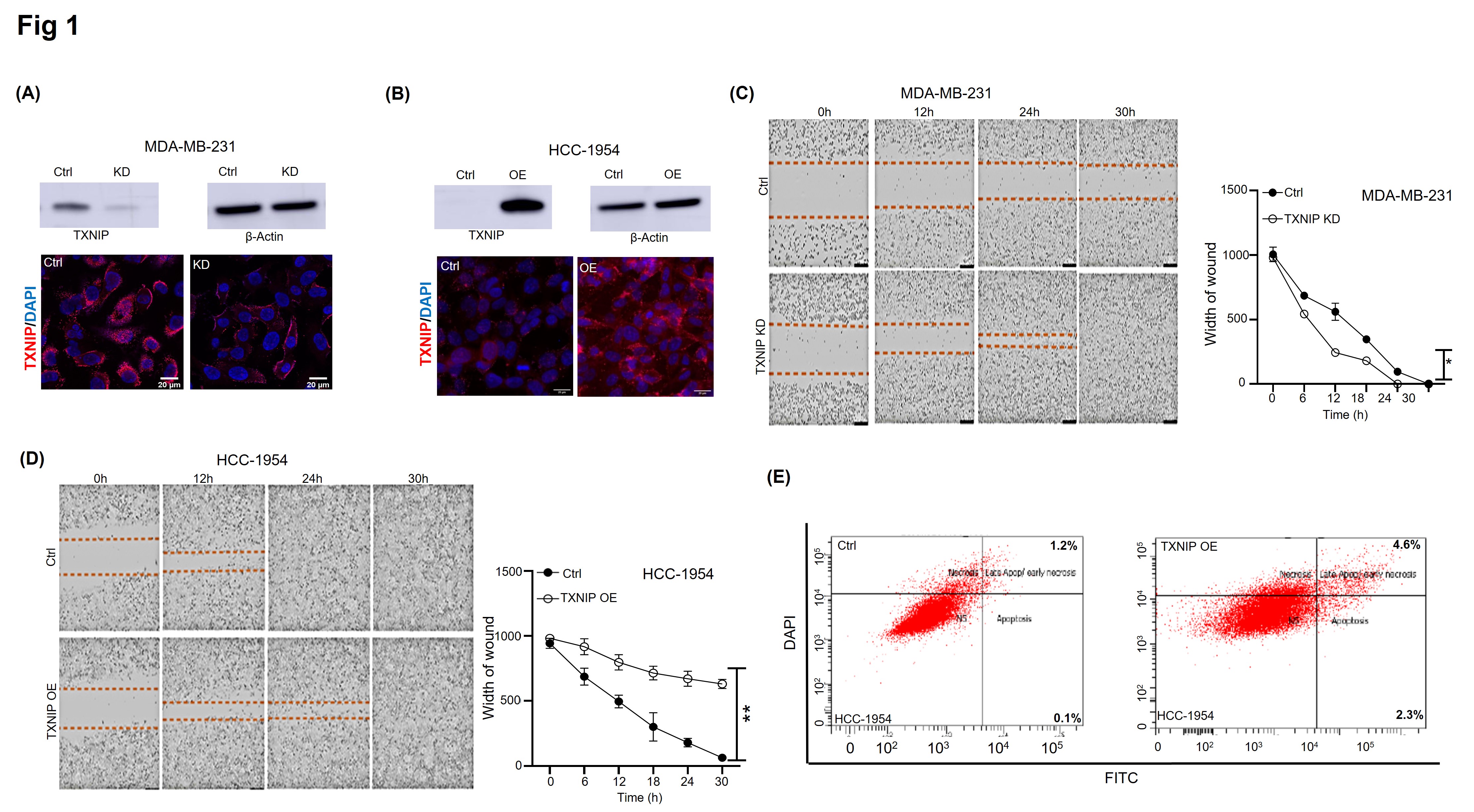

Supplement: Supplementary file 2 — Supplementary Figure 1 [file 41419_2025_7566_MOESM2_ESM.jpg]

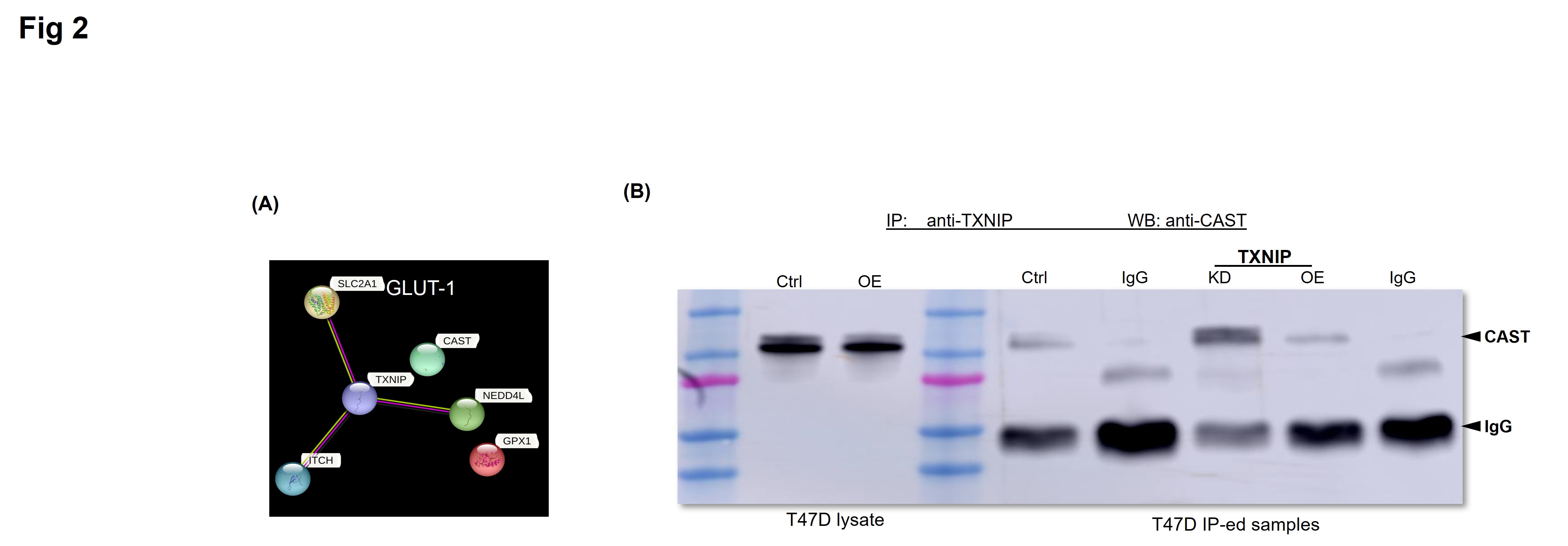

Supplement: Supplementary file 3 — Supplementary Figure 2 [file 41419_2025_7566_MOESM3_ESM.jpg]

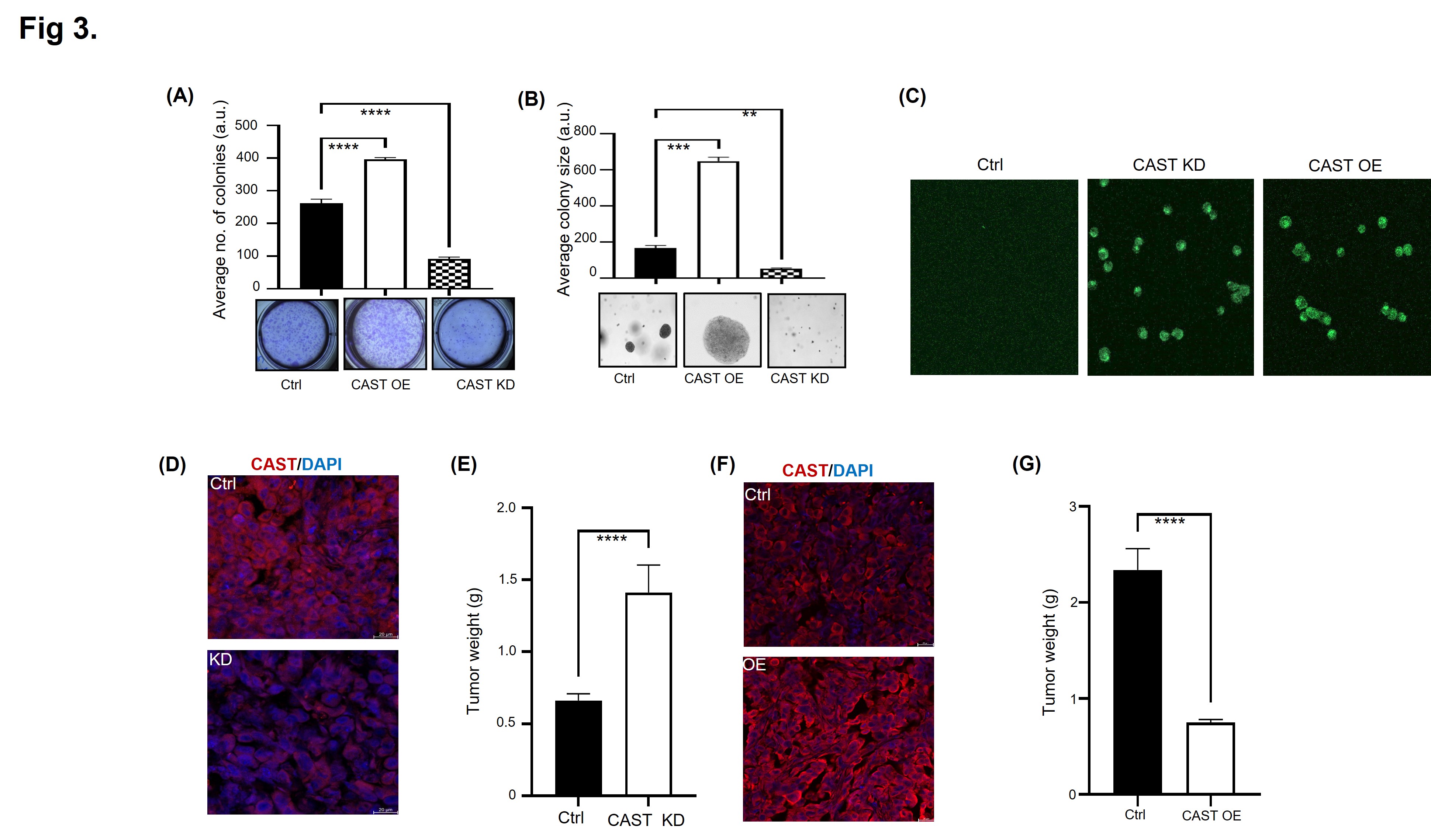

Supplement: Supplementary file 4 — Supplementary Figure 3 [file 41419_2025_7566_MOESM4_ESM.jpg]

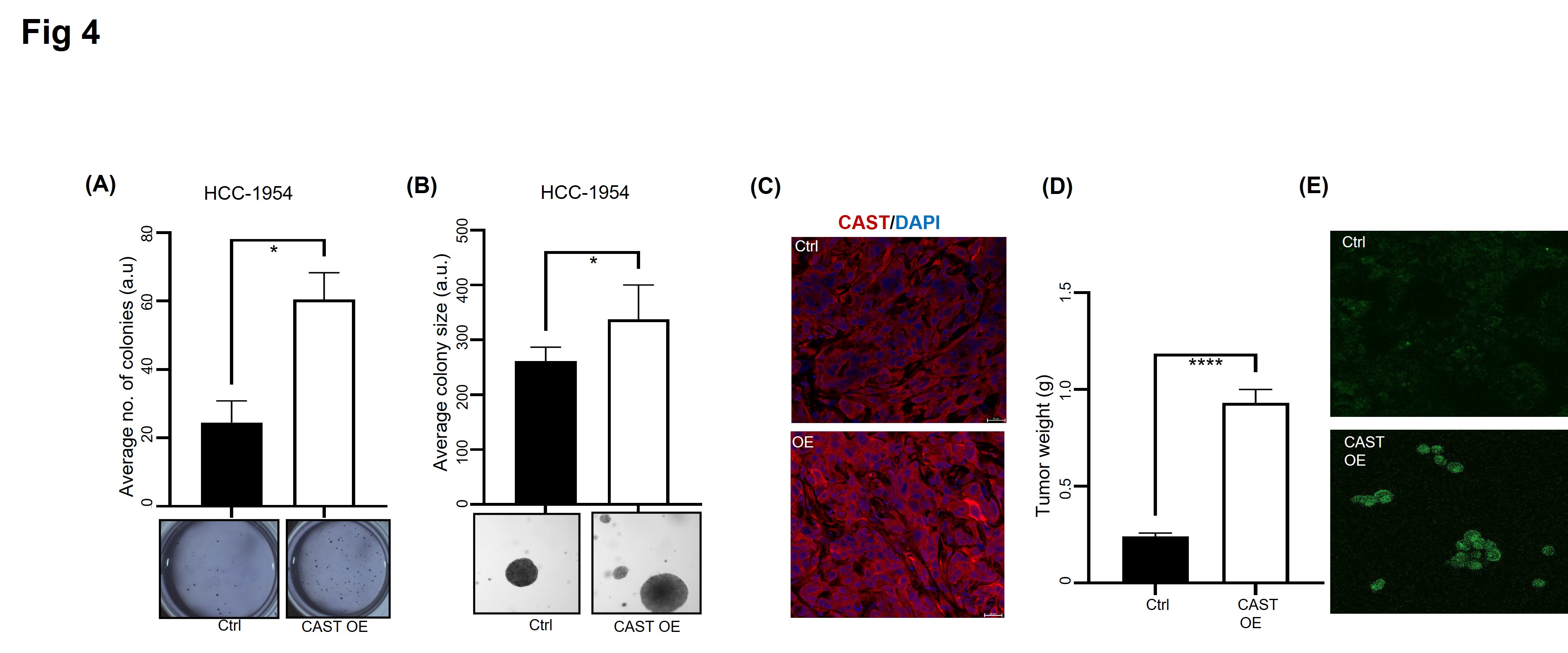

Supplement: Supplementary file 5 — Supplementary Figure 4 [file 41419_2025_7566_MOESM5_ESM.jpg]

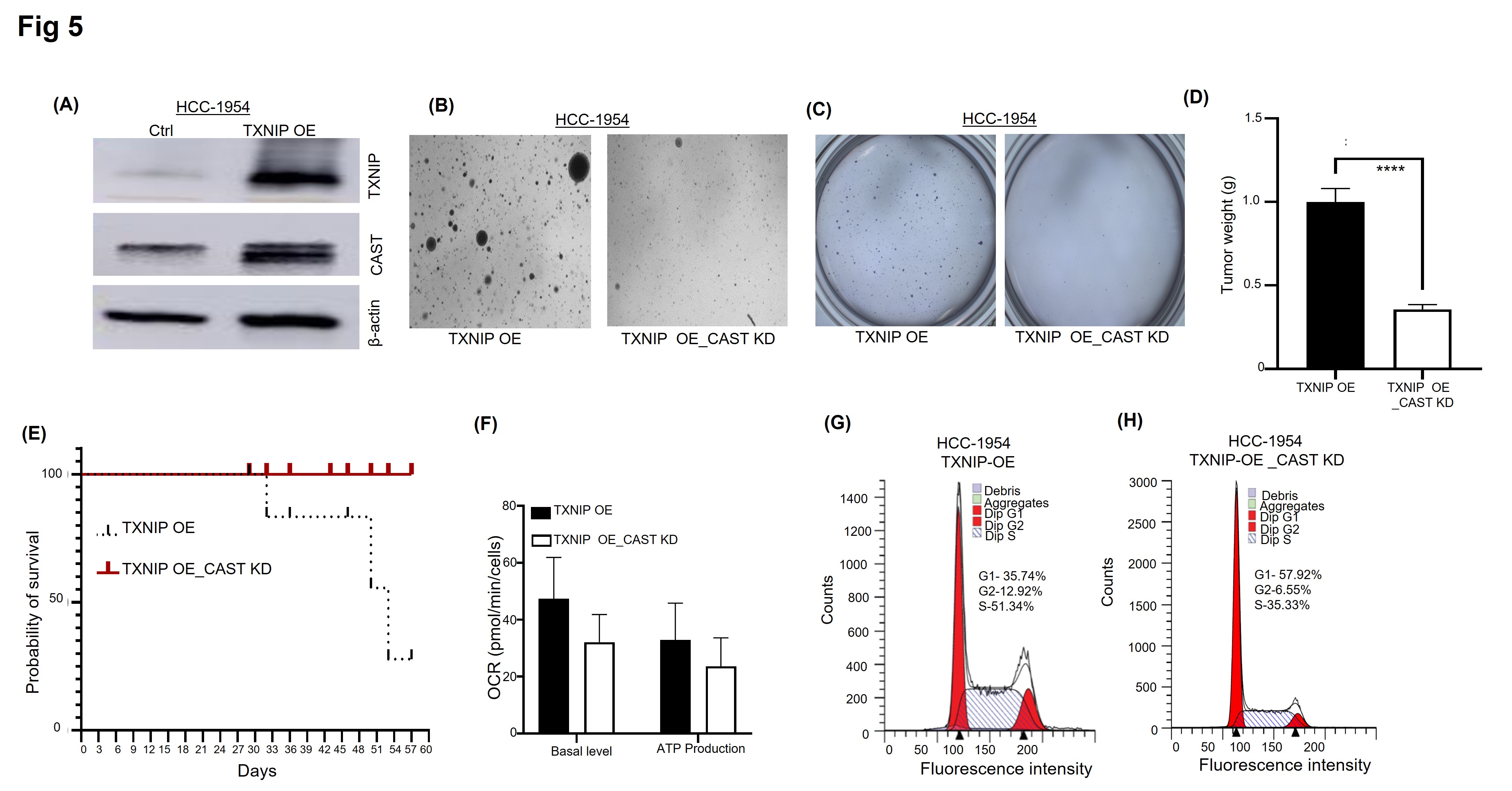

Supplement: Supplementary file 6 — Supplementary Figure 5-1 [file 41419_2025_7566_MOESM6_ESM.jpg]

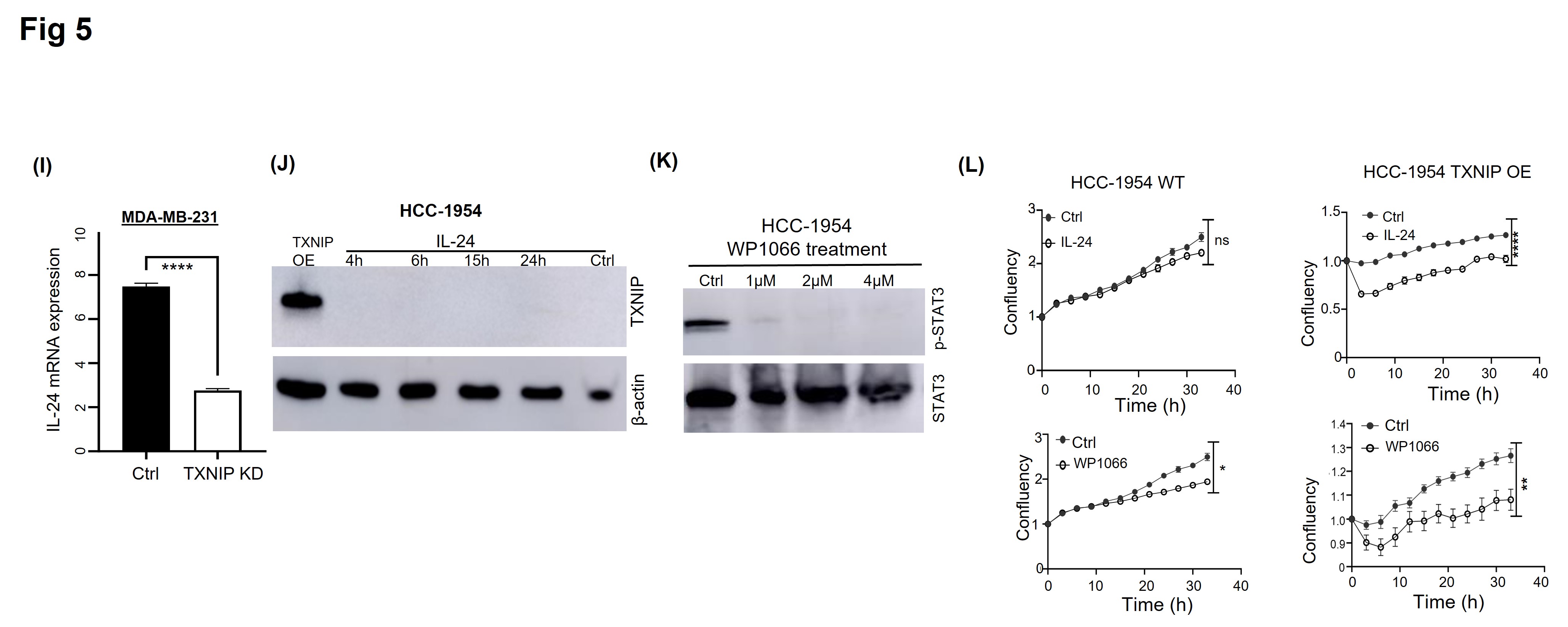

Supplement: Supplementary file 7 — Supplementary Figure 5-2 [file 41419_2025_7566_MOESM7_ESM.jpg]
