# Supplementary material for: Molecular mechanisms underlying TXNIP’s anti-tumor role in breast cancer, including interaction with a novel, pro-tumor partner: CAST
Source: Cell Death Dis. 2025 Apr 2;16(1):236. doi: 10.1038/s41419-025-07566-4 (PMC11965567; doi:10.1038/s41419-025-07566-4)
Supplement: Supplementary file 8 — Supplementary Table [file 41419_2025_7566_MOESM8_ESM.pptx]

## Slide 1
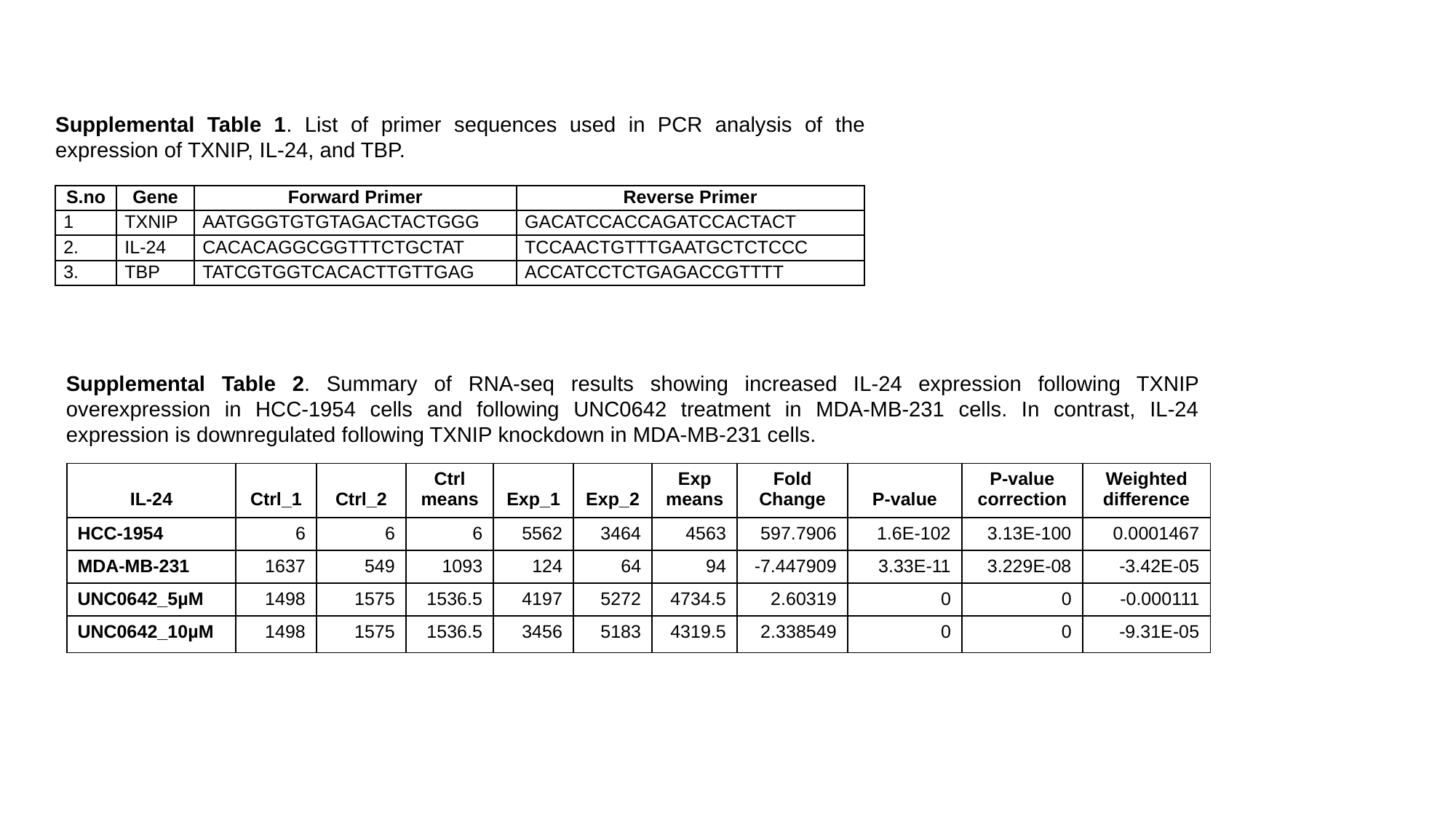

Supplemental Table 1. List of primer sequences used in PCR analysis of the expression of TXNIP, IL-24, and TBP.
| S.no | Gene | Forward Primer | Reverse Primer |
| --- | --- | --- | --- |
| 1 | TXNIP | AATGGGTGTGTAGACTACTGGG | GACATCCACCAGATCCACTACT |
| 2. | IL-24 | CACACAGGCGGTTTCTGCTAT | TCCAACTGTTTGAATGCTCTCCC |
| 3. | TBP | TATCGTGGTCACACTTGTTGAG | ACCATCCTCTGAGACCGTTTT |
Supplemental Table 2. Summary of RNA-seq results showing increased IL-24 expression following TXNIP overexpression in HCC-1954 cells and following UNC0642 treatment in MDA-MB-231 cells. In contrast, IL-24 expression is downregulated following TXNIP knockdown in MDA-MB-231 cells.
| IL-24 | Ctrl\_1 | Ctrl\_2 | Ctrl means | Exp\_1 | Exp\_2 | Exp means | Fold Change | P-value | P-value correction | Weighted difference |
| --- | --- | --- | --- | --- | --- | --- | --- | --- | --- | --- |
| HCC-1954 | 6 | 6 | 6 | 5562 | 3464 | 4563 | 597.7906 | 1.6E-102 | 3.13E-100 | 0.0001467 |
| MDA-MB-231 | 1637 | 549 | 1093 | 124 | 64 | 94 | -7.447909 | 3.33E-11 | 3.229E-08 | -3.42E-05 |
| UNC0642\_5µM | 1498 | 1575 | 1536.5 | 4197 | 5272 | 4734.5 | 2.60319 | 0 | 0 | -0.000111 |
| UNC0642\_10µM | 1498 | 1575 | 1536.5 | 3456 | 5183 | 4319.5 | 2.338549 | 0 | 0 | -9.31E-05 |
