# Supplementary material for: Molecular mechanisms underlying TXNIP’s anti-tumor role in breast cancer, including interaction with a novel, pro-tumor partner: CAST
Source: Cell Death Dis. 2025 Apr 2;16(1):236. doi: 10.1038/s41419-025-07566-4 (PMC11965567; doi:10.1038/s41419-025-07566-4)

Uncropped blot for Fig.3

(B)

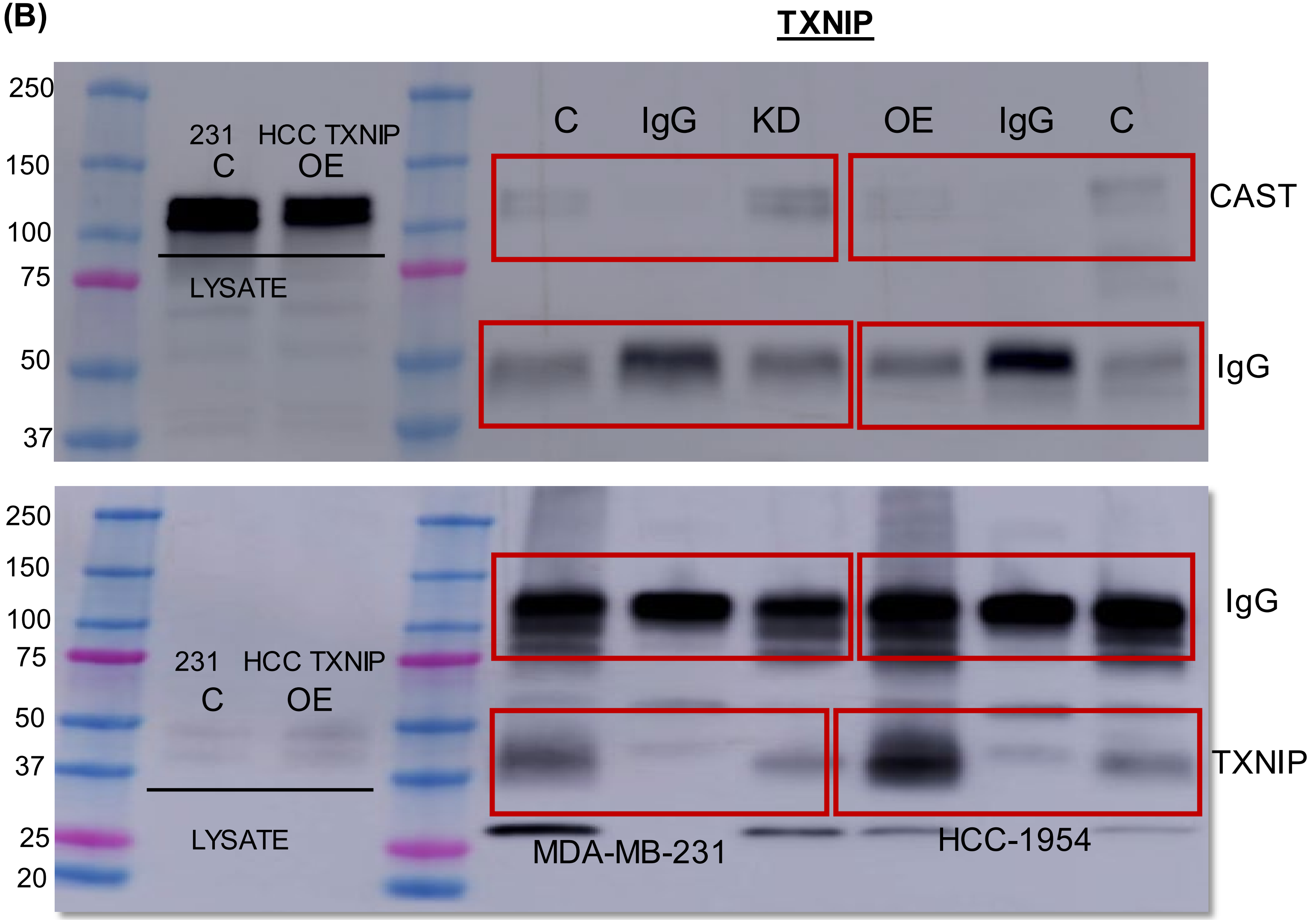

I.P anti-TXNIP

Uncropped blot for Fig.3

(B)

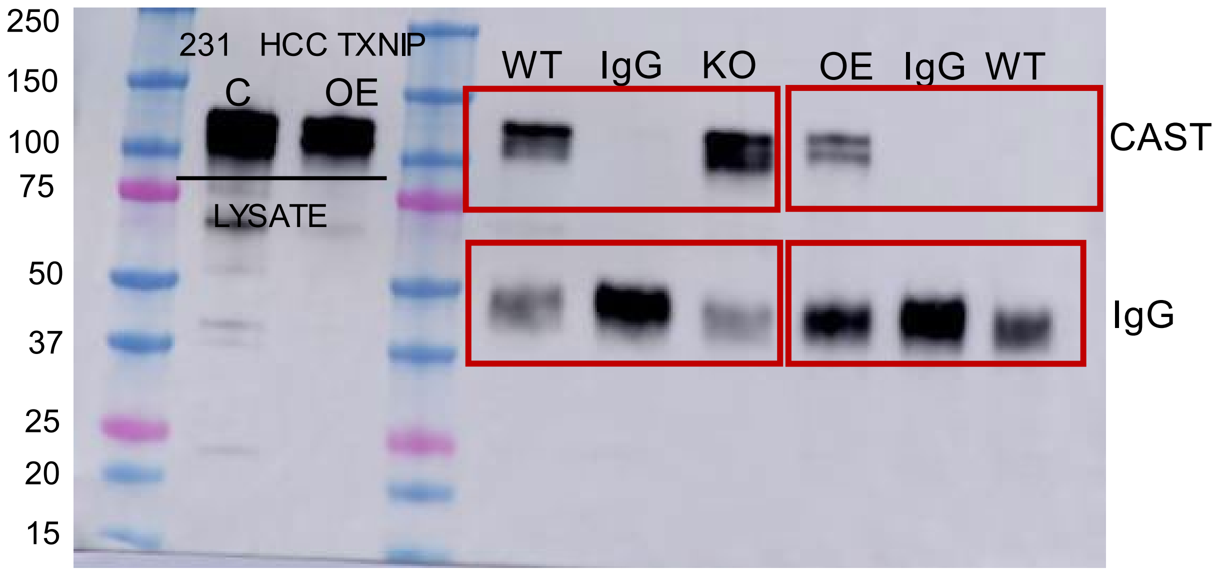

I.P anti-CAST

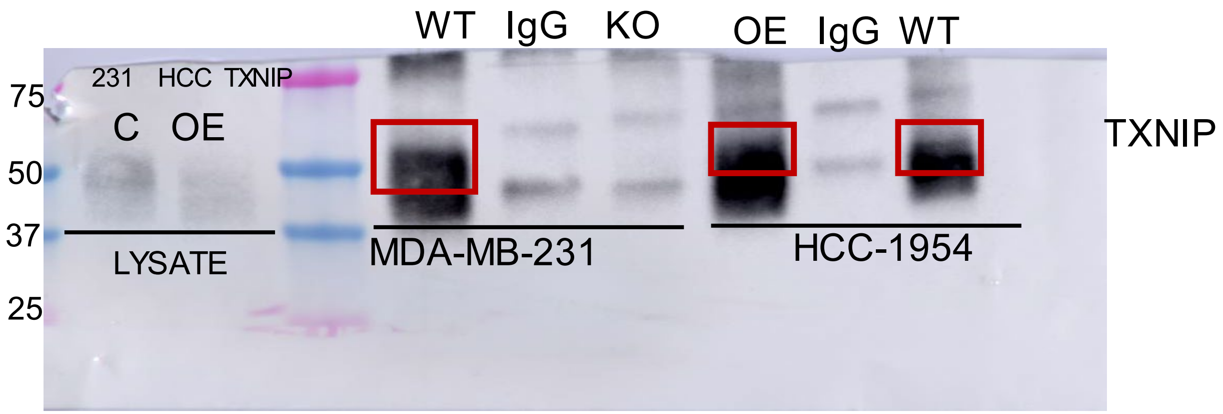

Uncropped blot for Fig.3

(D)

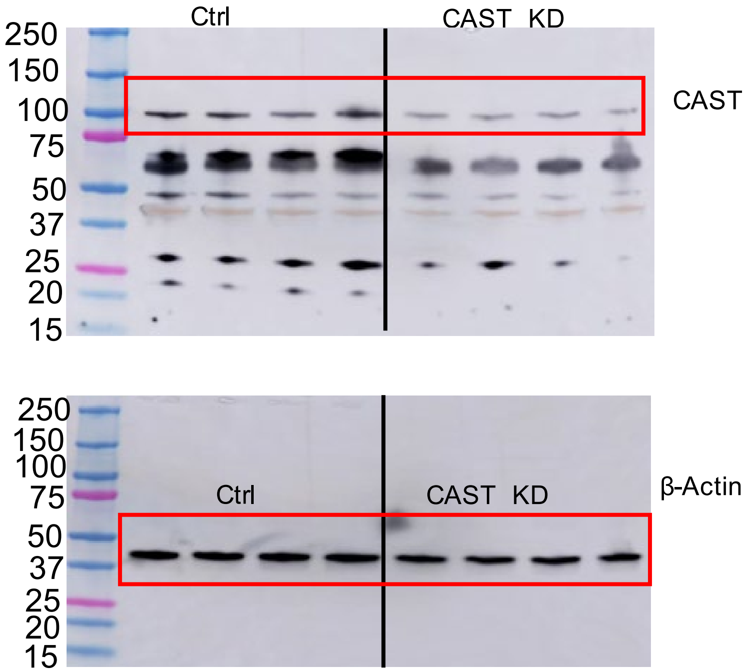

(E)

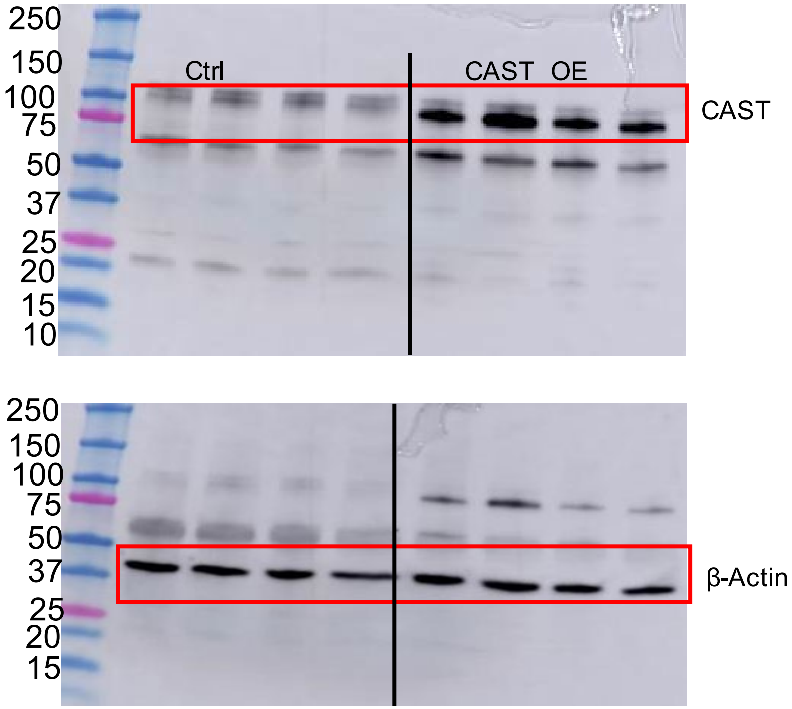

Uncropped blot for Fig.4

(B)

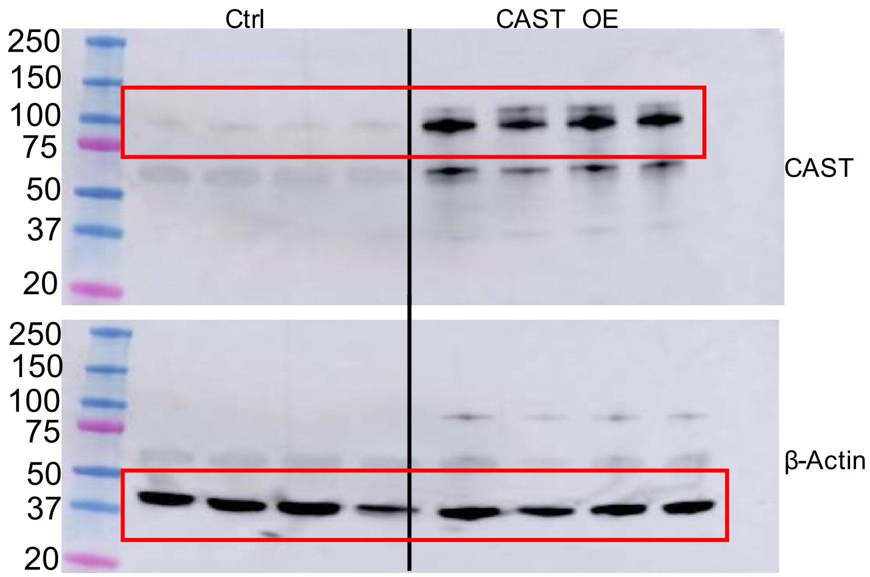

(D)

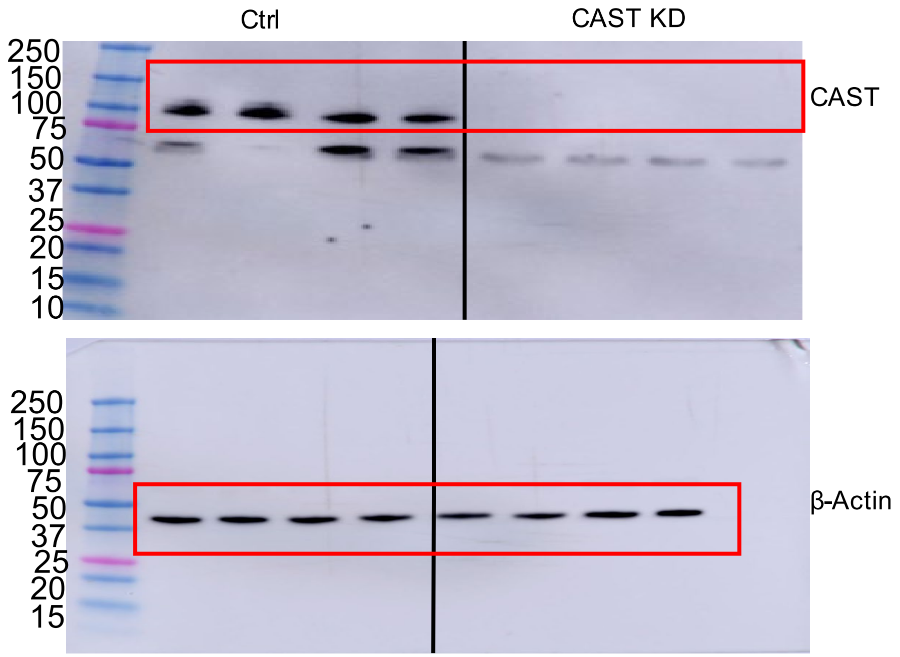

Uncropped blot for Fig.5

(C)

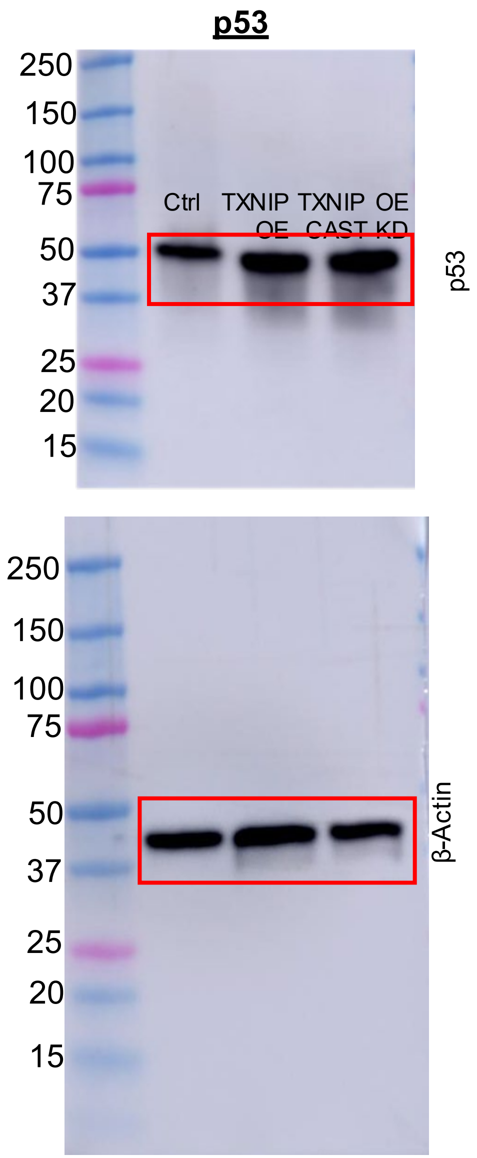

(D)

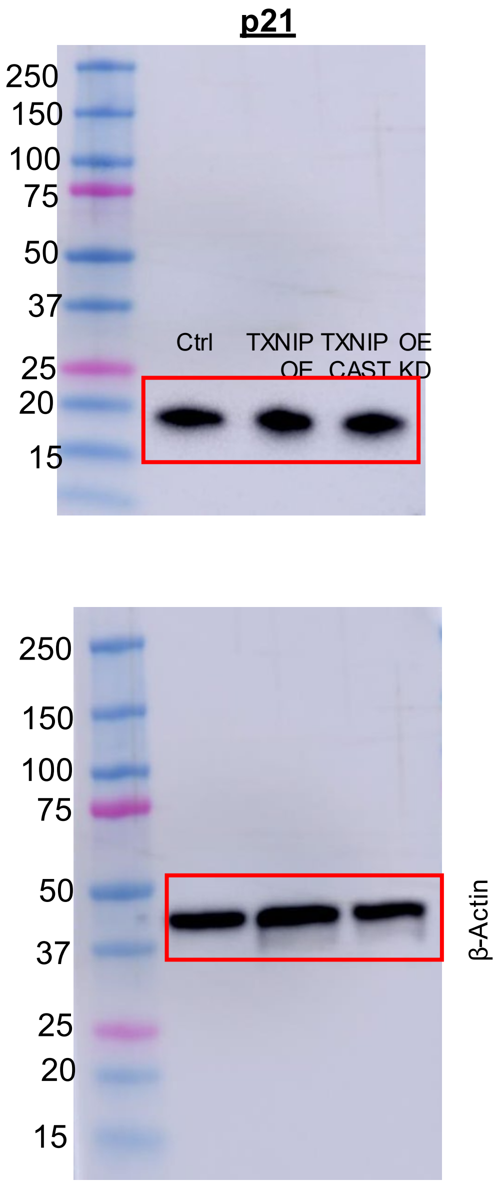

(E)

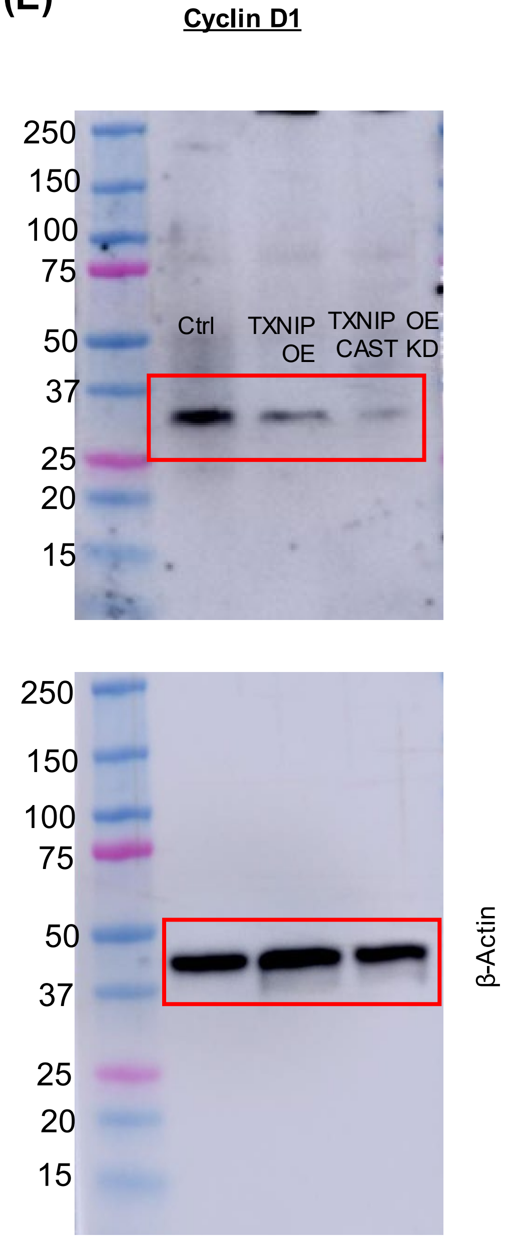

Uncropped blot for Fig.6

(E)

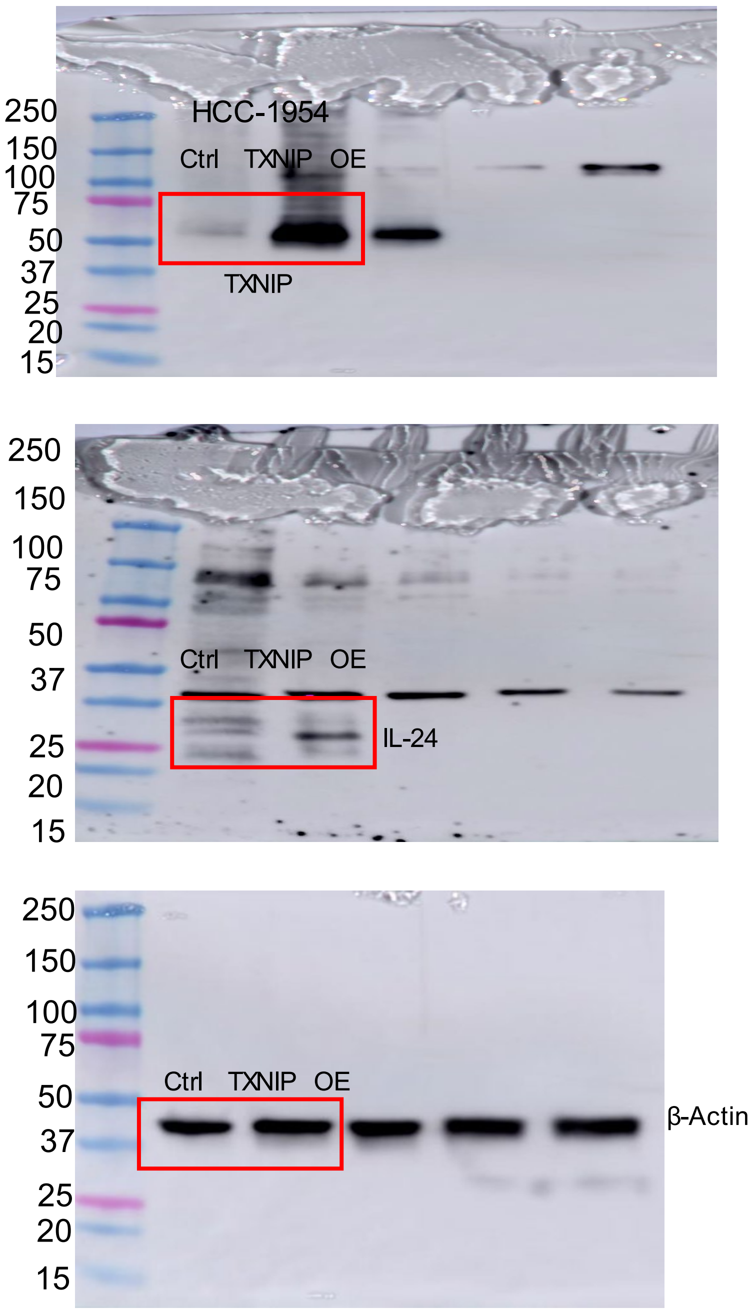

(E)

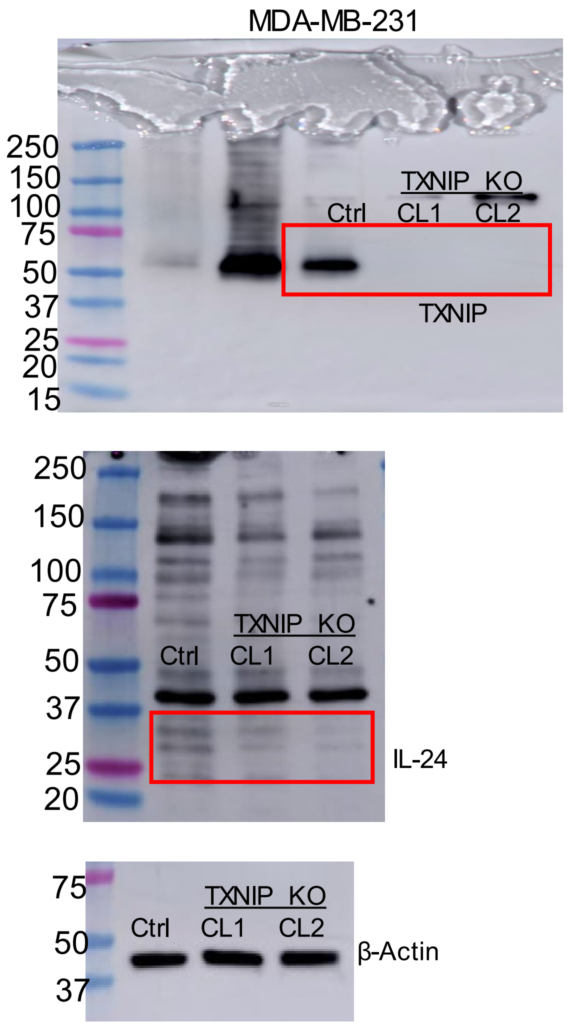

Uncropped blot for Fig.6

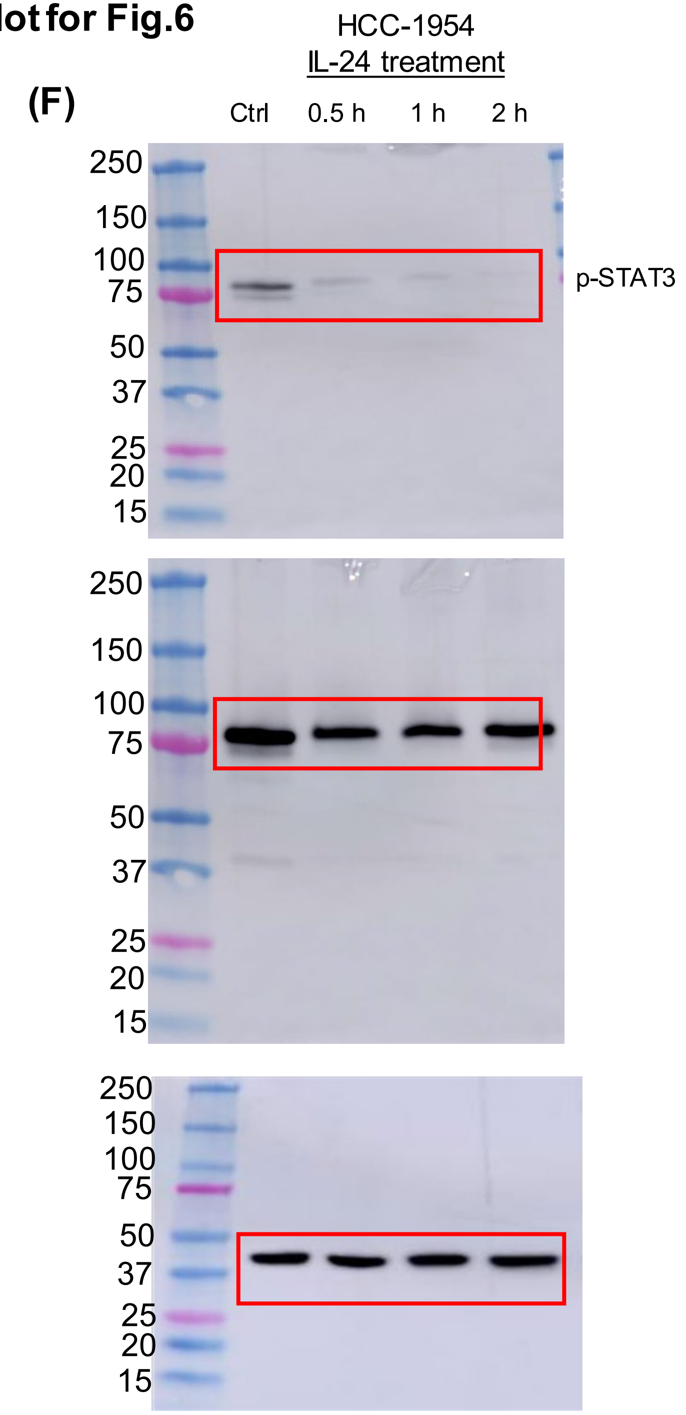

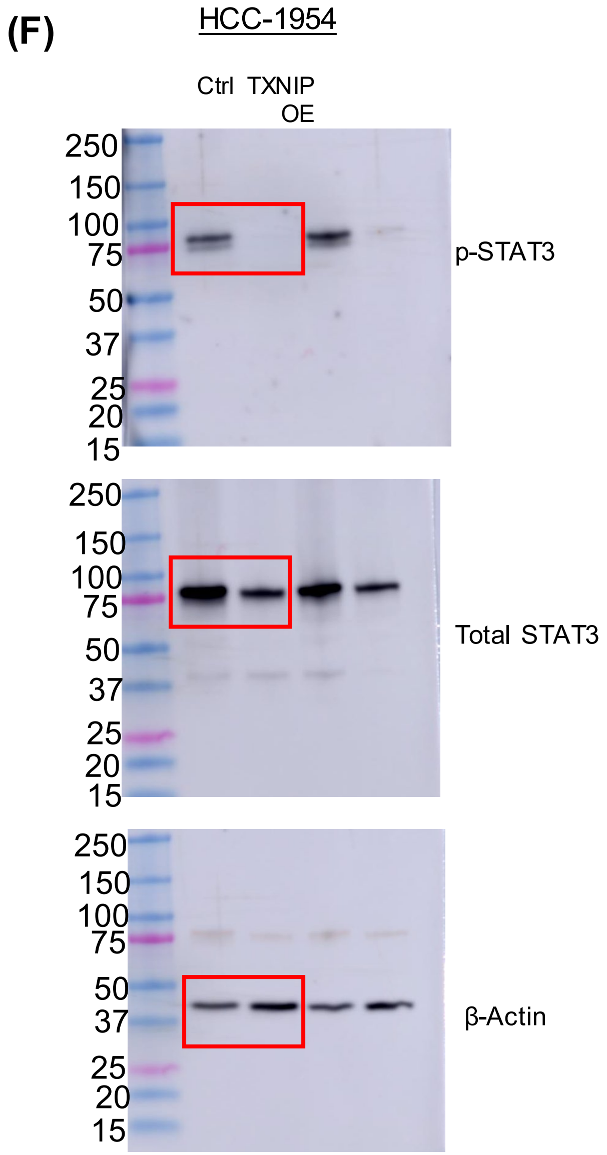

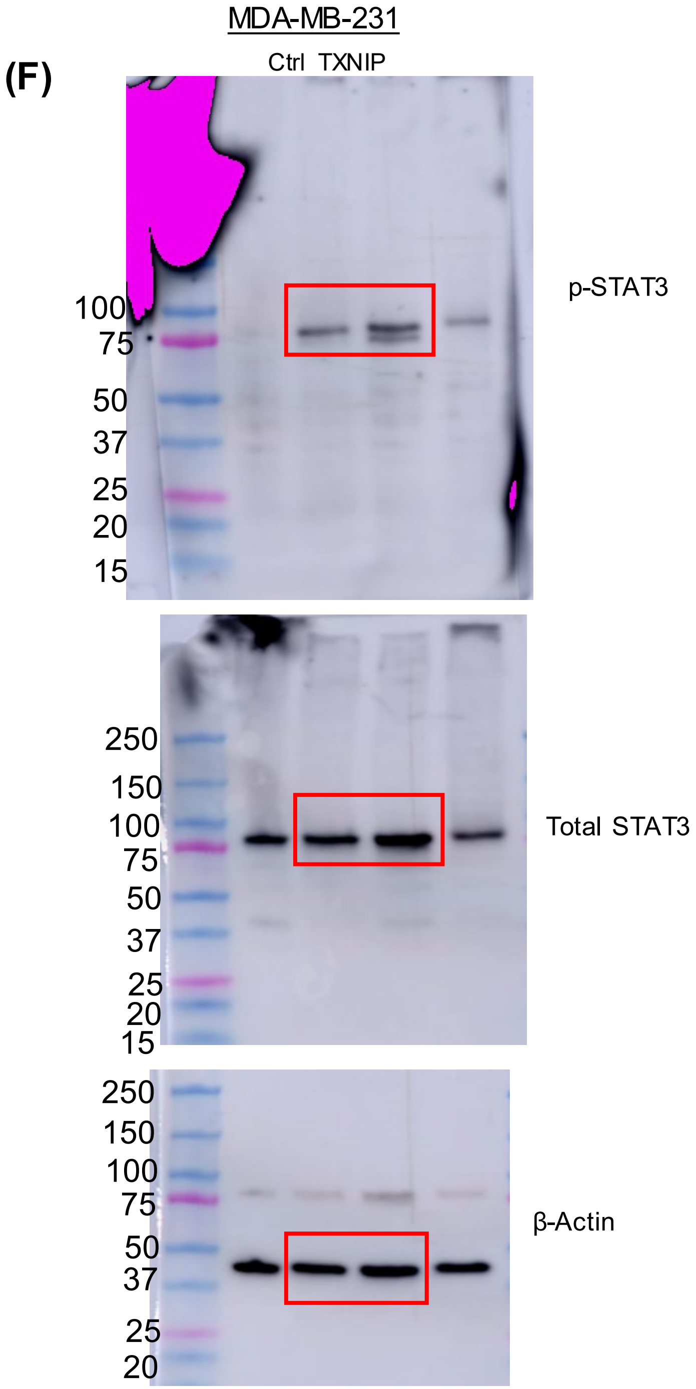

Uncropped blot for supplementary Fig.1

(A)

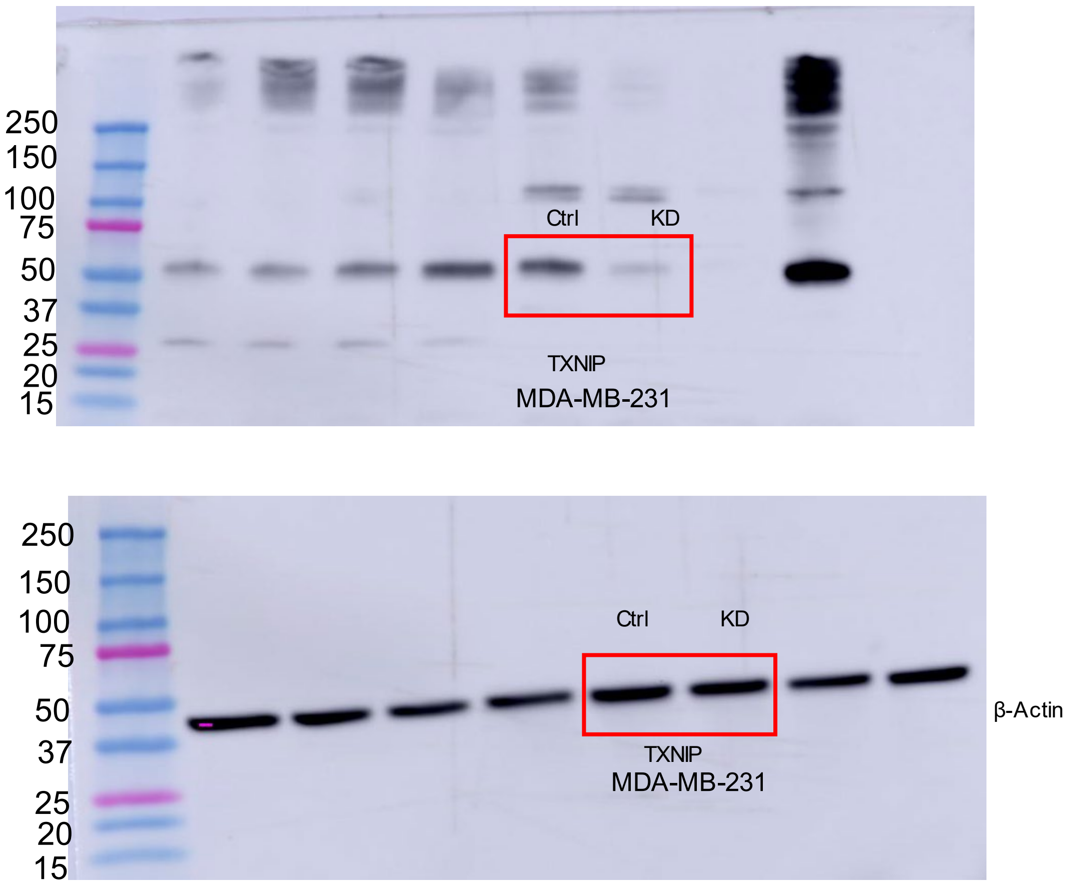

(b)

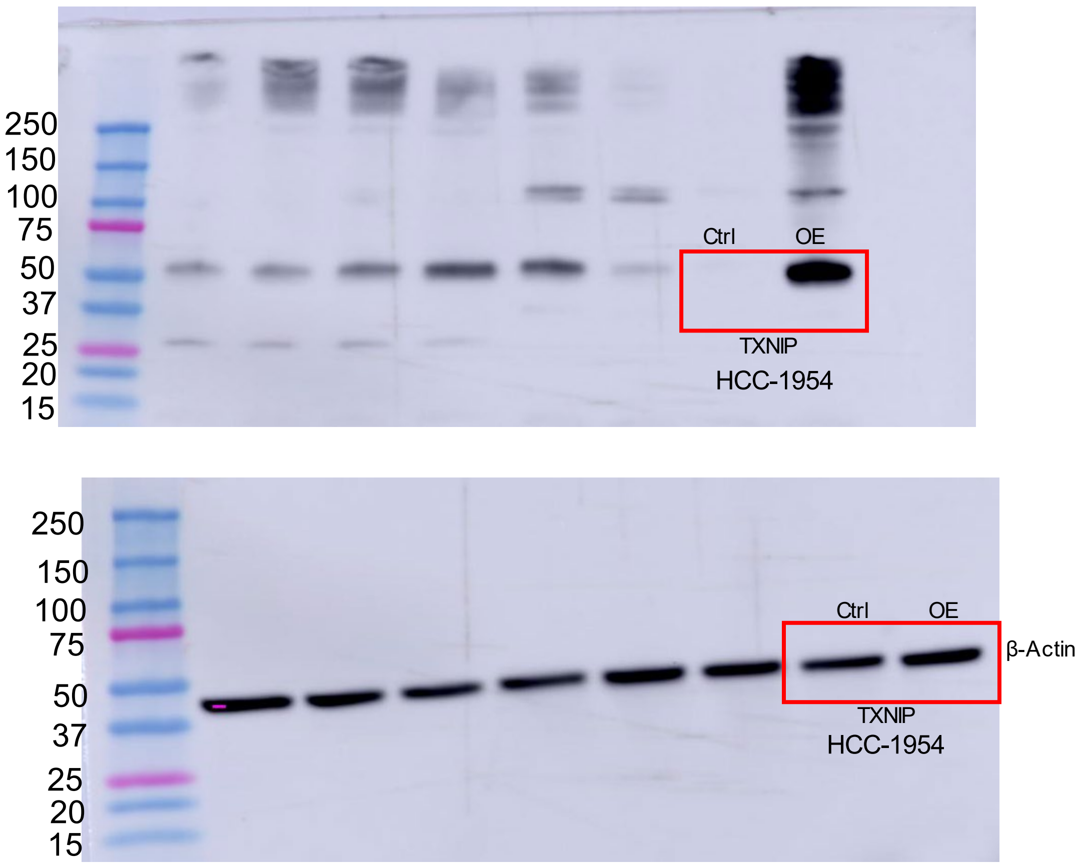

Uncropped blot for supplementary Fig.2

(B)

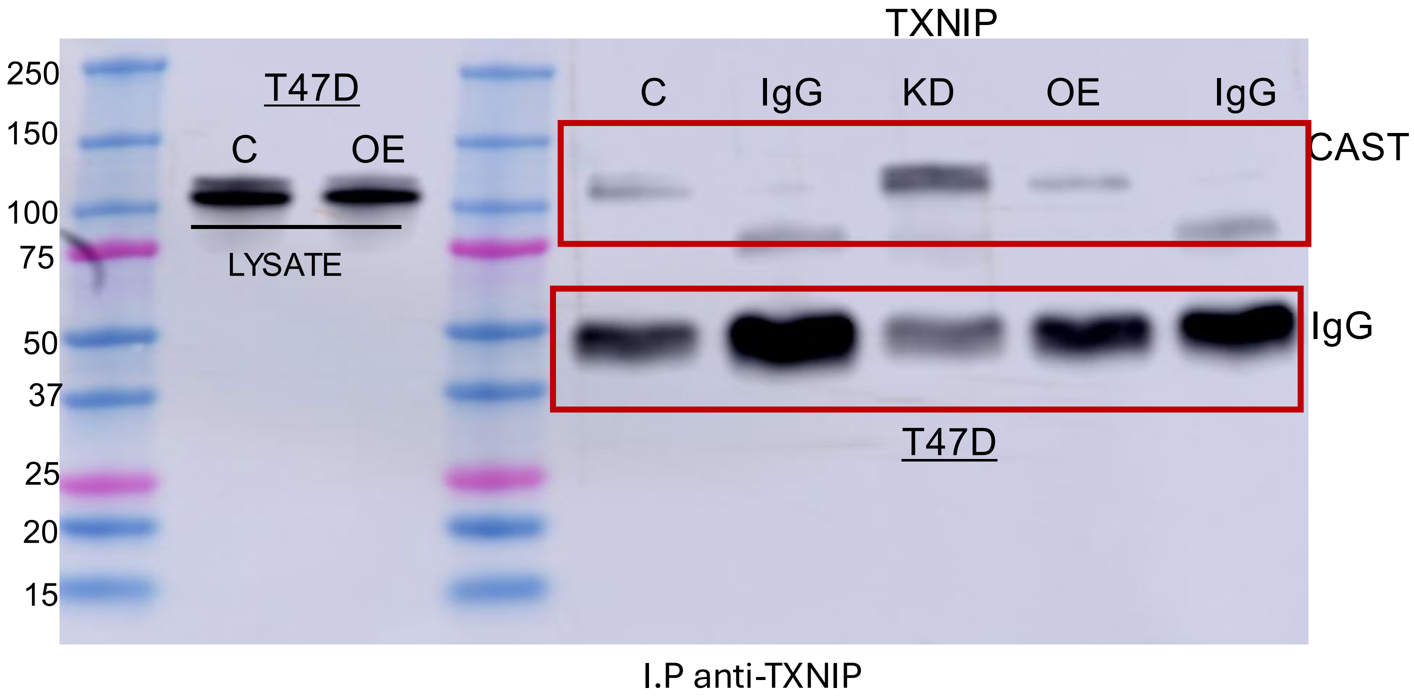

Uncropped blot for supplementary Fig.5

(A)

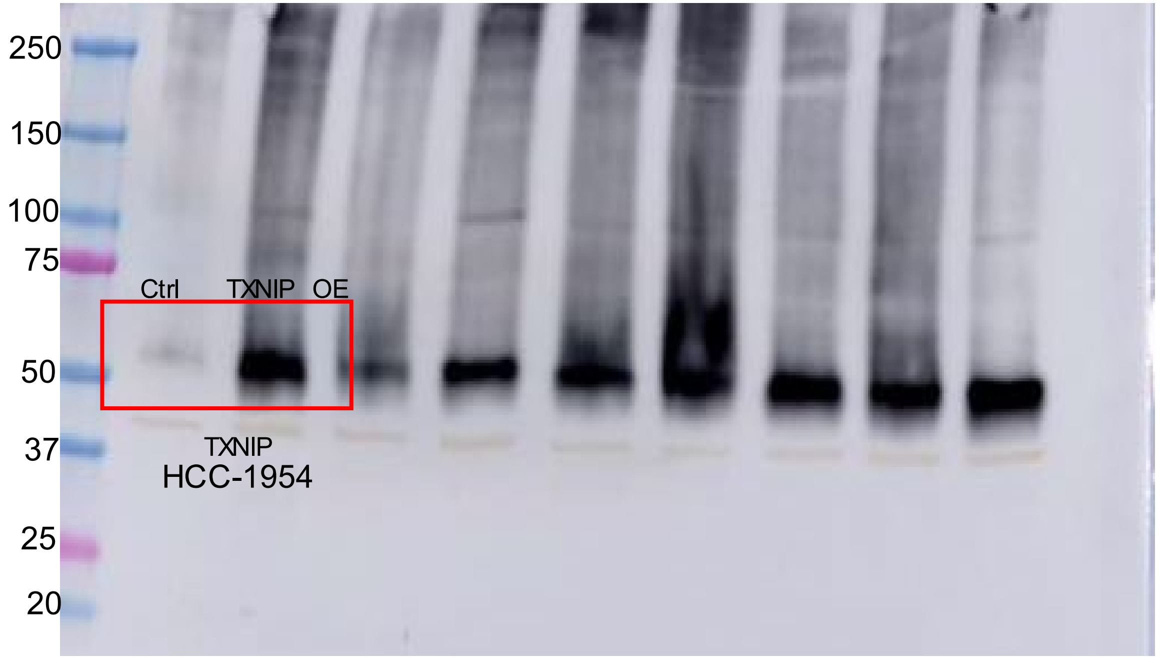

(A)

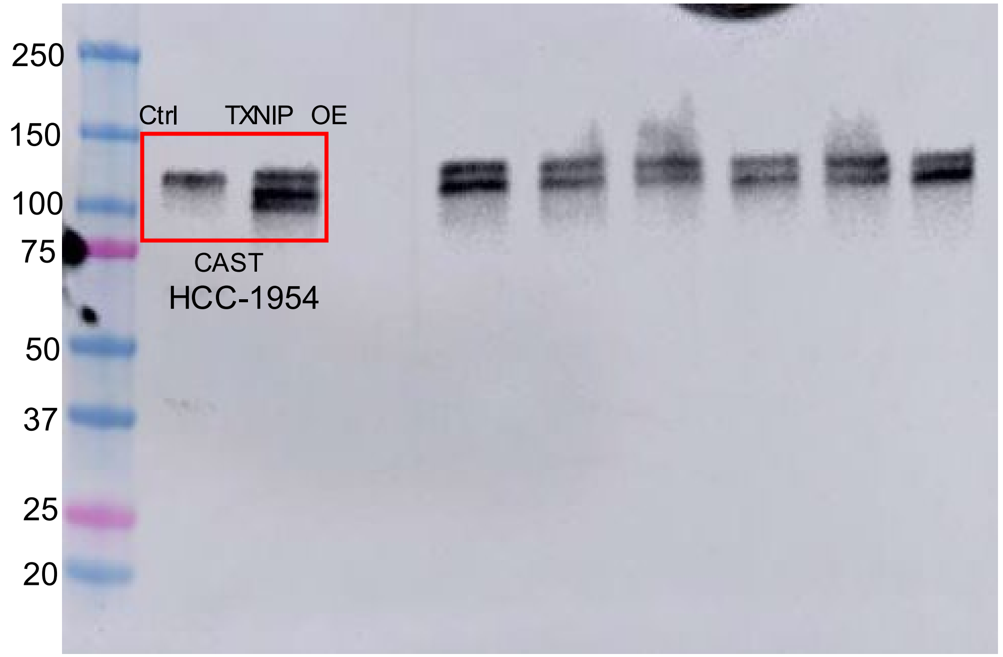

(A)

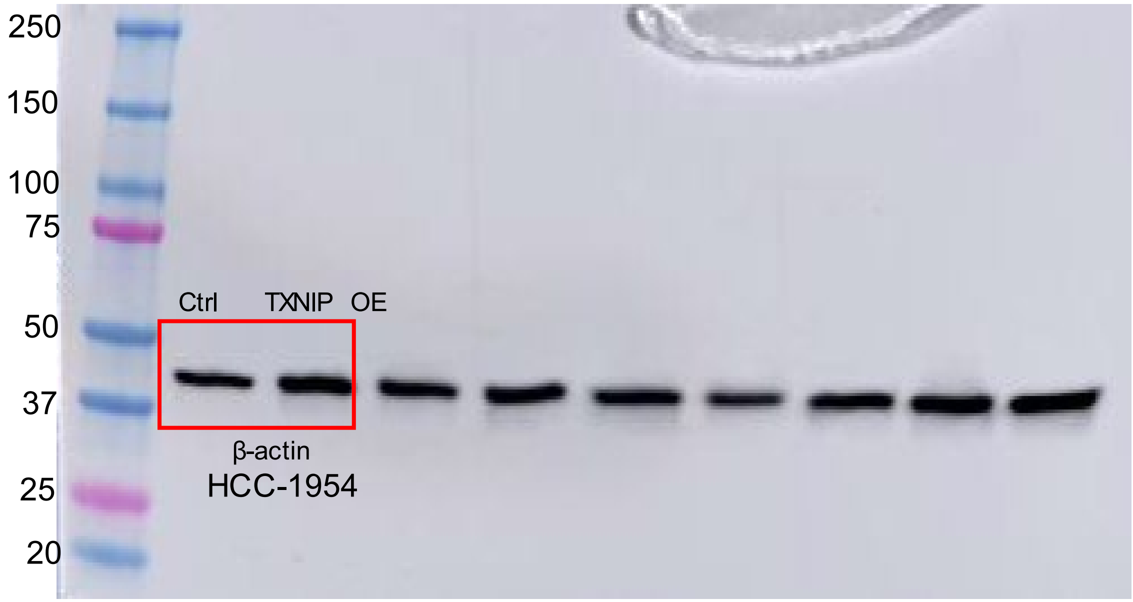

Uncropped blot for supplementary Fig.5

(I)

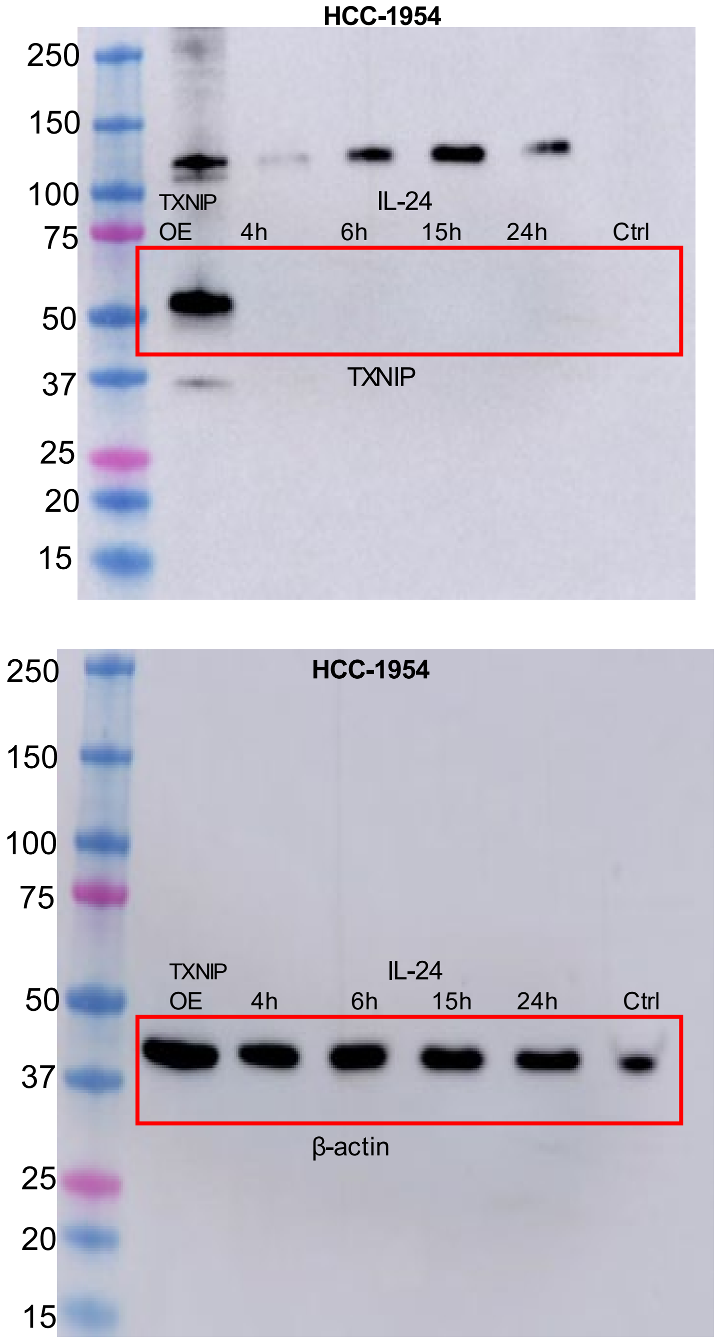

(J)

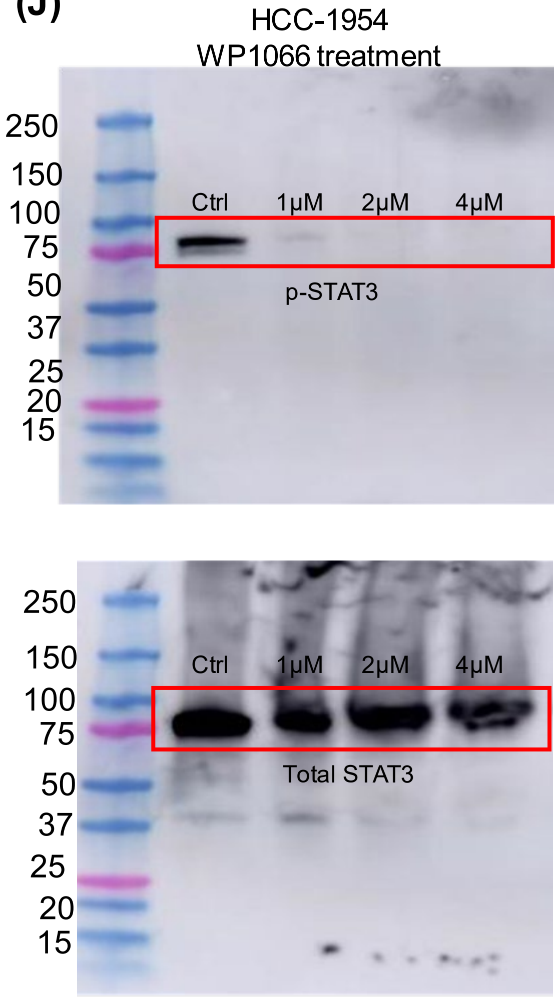

Supplement: Supplementary file 9 — Orginal Western Blots [file 41419_2025_7566_MOESM9_ESM.pdf]
